# GABAergic Modulation of Brain Function During Prosaccade and Antisaccade Eye Movements: Evidence From Ultra‐High‐Field fMRI

**DOI:** 10.1002/hbm.70598

**Published:** 2026-07-05

**Authors:** Paulina Quint, Pia‐Magdalena Schmidt, Sarah Mackert, Behrem Aslan, Matthias Guth, Leon von der Emde, Raphael Lechtenboehmer, Kaja Faßbender, Philine M. Baumert, Birgit Stoffel‐Wagner, Ramona Dolscheid‐Pommerich, Rüdiger Stirnberg, Tony Stöcker, Ulrich Ettinger

**Affiliations:** ^1^ Department of Psychology University of Bonn Bonn Germany; ^2^ Department of Psychiatry and Psychotherapy University Hospital Bonn Bonn Germany; ^3^ Department of Ophthalmology University Hospital Bonn Bonn Germany; ^4^ Institute of Clinical Chemistry and Clinical Pharmacology University Hospital Bonn Bonn Germany; ^5^ German Center for Neurodegenerative Diseases (DZNE) Bonn Germany

**Keywords:** benzodiazepines, eye‐tracking, fMRI, GABA_A_ receptor, inhibitory control, saccades

## Abstract

Benzodiazepines act as positive allosteric modulators of the GABA_A_ receptor and affect several motor and cognitive functions. By engaging perceptual‐motor as well as inhibitory control processes, the antisaccade task was used in previous studies to investigate effects of benzodiazepines on behavioral performance. Using a randomized, double‐blind, placebo‐controlled within‐subjects design, this study combined eye‐tracking with BOLD fMRI in order to examine the neural correlates of these effects for the first time. *N* = 39 healthy participants completed an antisaccade task after administration of either 1 mg lorazepam or placebo. On a behavioral level, lorazepam led to reduced (anti‐)saccadic peak velocity as well as increased (anti‐)saccadic latency. On a neural level, drug‐induced reduction of BOLD was found in a fronto‐parietal‐occipital network, including key oculomotor regions. This result was further supported by our finding of increased GABA_A_ receptor density in the affected network. On an individual level, decline in peak velocity under lorazepam was associated with decreased neural activation in several cortical regions, including medial frontal eye fields. No interactions between drug and saccade condition (prosaccade, antisaccade) were found. Our results therefore suggest GABAergic modulation of a more general saccade‐related network rather than of specific components for inhibitory control processes. Future studies may rely on BOLD signal as a sensitive marker for benzodiazepine activity during saccadic eye movements.

## Summary

- Lorazepam led to reduced prosaccade and antisaccade peak velocity, increased latency, and decreased activation in a frontal–parietal–occipital oculomotor network.
- This lorazepam‐affected network showed higher GABA_A_ receptor density than other brain areas.
- Reduced fronto‐parieto‐occipital activation due to lorazepam was associated with individual decline in saccadic peak velocity.

## Graphical abstract

Combining eye‐tracking with 7‐Tesla fMRI, we found that lorazepam impacted (anti‐)saccadic eye movements and reduced BOLD in a fronto‐parietal‐occipital network. These areas showed higher GABA_A_ receptor density than remaining brain areas. Individual peak velocity reductions under lorazepam were linked to lower activation in cortical regions, including medial frontal eye fields.

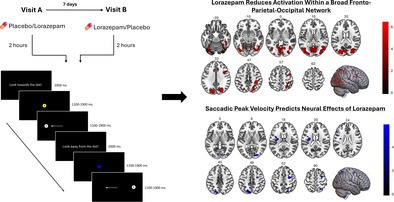

## 1 Introduction

Benzodiazepines (BZD) are effective medications for several indications, including anxiety disorders and epileptic seizures. They act as positive allosteric modulators of the GABA_A_ receptor (Baldwin et al. 2013; Cloos and Ferreira 2009) and affect motor and cognitive functions, after both acute and long‐term use (Chen et al. 2015; Crowe and Stranks 2018; De Visser et al. 2003). One of the most consistent, acute effects of BZD is a reduction in saccadic peak velocity (Baumert et al. 2024; De Haas et al. 2007; Schmidt et al. 2026), with this measure therefore being used as a biomarker for BZD activity (De Visser et al. 2003). Further, BZD have been associated with acute impairments in cognitive control, including attentional shifting (Boucart et al. 2007), updating (Forsyth et al. 2021) and selective attention (Faßbender et al. 2023). Impairments also affect inhibitory control, i.e., the ability to suppress dominant or automatic responses and to resist interference from distractors (Friedman and Miyake 2004). Across different paradigms, studies have demonstrated effects of BZD on inhibitory control that extend beyond psychomotor slowing (Fillmore et al. 2001; Sarkar et al. 2020; Schunck et al. 2010). Relying on the advantages of oculography such as high precision and well‐known neural mechanisms (Reilly et al. 2008), studies have used the antisaccade task to investigate inhibitory control following BZD administration.

In the antisaccade task, participants are instructed to not look at a sudden‐onset, peripheral target, but in the opposite direction instead. It imposes demands on fundamental perceptual‐motor and higher cognitive processes, including inhibition of an automatic saccade toward the target, vector inversion and generation of a saccade in the opposite direction (Hutton 2008; Munoz and Everling 2004). Previous studies found increased antisaccade latency and error rate (Faßbender et al. 2021; Green et al. 2000; Green and King 1998) as well as decreased antisaccade peak velocity (McCartan et al. 2001) after BZD administration.

The neural network underlying antisaccades is well studied and encompasses frontal, parietal, occipital and subcortical areas (Cieslik et al. 2016; Ettinger et al. 2008; McDowell et al. 2008). In addition to prosaccade‐related regions, increased activation during antisaccades occurs in frontal eye fields (FEF), supplementary eye fields (SEF) and intraparietal sulcus (IPS; Brown et al. 2006; Ettinger et al. 2008; Jamadar et al. 2013; Kimmig et al. 2001), which can be attributed to preparation and generation of a voluntary saccade and spatial vector inversion, respectively (Connolly et al. 2002; Herweg et al. 2014; McDowell et al. 2008). Further relevant areas include dorsolateral prefrontal cortex (DLPFC) and anterior cingulate cortex (ACC; Brown et al. 2006; Jamadar et al. 2013; Ploner et al. 2005). Given this well‐known neural circuitry, the task lends itself ideally to characterizing BZD effects on brain function during inhibitory control.

However, despite a wealth of neuroimaging studies on antisaccades and other inhibitory control tasks (Jamadar et al. 2013; Zhang et al. 2017), underlying neurotransmitter systems are less well characterized. Previous studies mainly focused on dopamine, noradrenaline, and acetylcholine (Ettinger et al. 2017; Logue and Gould 2014; Robbins and Arnsten 2009) and have not yet adequately addressed the role of inhibitory neurotransmitters. Against the background of regional correlations between cerebral blood flow and neurotransmitter activity (Dukart et al. 2018), we explored the neural mechanisms of GABA‐modulations of antisaccade performance by combining functional magnetic resonance imaging (fMRI) at ultra‐high‐field strength (7 T) with eye‐tracking after administration of 1 mg lorazepam, one of the most widely researched and clinically prescribed BZD.

In this preregistered study (https://osf.io/by6xu/overview?view_only=69bcc676c02a4253bb3f4971e527819c), we expected to replicate effects of lorazepam on (anti‐)saccadic peak velocity, latency, and error rate (Faßbender et al. 2021; McCartan et al. 2001), and we expected neural effects to be located in task‐related regions (Brown et al. 2006; Cieslik et al. 2016; Ettinger et al. 2008; Jamadar et al. 2013). We further investigated lorazepam effects on functional connectivity and explored neural correlates of individual differences in lorazepam response. Finally, to shed light on the molecular basis of the observed effects, we explored whether the magnitude of lorazepam effects on brain function covaried with regional differences in GABA_A_ receptor density (Nørgaard et al. 2021).

## 2 Materials and Methods

Additional details on the study procedure and data analyses can be found in the Supporting Information Methods.

### 2.1 Sample and Study Design

A sample size of 40 healthy participants, aged 18–40 years was targeted to provide approximately 90% power to detect an effect with an estimated *d* = 0.5 (Ettinger et al. 2018) at an alpha error probability of 0.05 (two‐tailed; G*Power; Faul et al. 2009). Complete inclusion and exclusion criteria are in the Supporting Information Methods. A randomized, double‐blind, placebo‐controlled within‐subjects design was employed. Each participant received placebo during one MRI session and 1 mg lorazepam during the other (order randomized across participants). The study was approved by the Ethics Committee of the Medical Faculty at the University of Bonn (No. 127/22) and conducted in accordance with the Declaration of Helsinki.

### 2.2 Study Procedure

Participants' study eligibility was confirmed via an online questionnaire and a detailed in‐person screening at the University of Bonn, where participants provided written, informed consent and inclusion and exclusion criteria were checked. Eligible participants were invited to take part in two MRI sessions at the German Center for Neurodegenerative Diseases (DZNE), Bonn, separated by approx. 1 week (median = 7 days, range 6–98). The procedure of both sessions was identical, except for the drug condition. After arrival, participants underwent alcohol and, for females only, pregnancy testing and were informed about MRI‐related safety aspects. Afterwards, a capsule containing either 1 mg lorazepam (Tavor, Pfizer) or a placebo (P‐Tabletten weiß 7 mm Lichtenstein, Zentiva) was administered, followed by a waiting period of 80 min, to allow task onset 2 h after administration (Saari et al. 2011). The scanning procedure started with a structural T1 scan, followed by three oculomotor tasks. The current task was performed first, approximately 120 min after capsule administration (*M* = 119 min, SD = 9 min). Remaining tasks will be reported elsewhere. Total scan time was about 1 h. After MRI, participants completed online visual analogue rating scales (VAS) assessing current mood state (alertness, contentedness and calmness; Bond and Lader 1974). Afterwards, blood pressure and pulse were measured and a blood sample was collected.

### 2.3 Saccade Task

**FIGURE 1:**
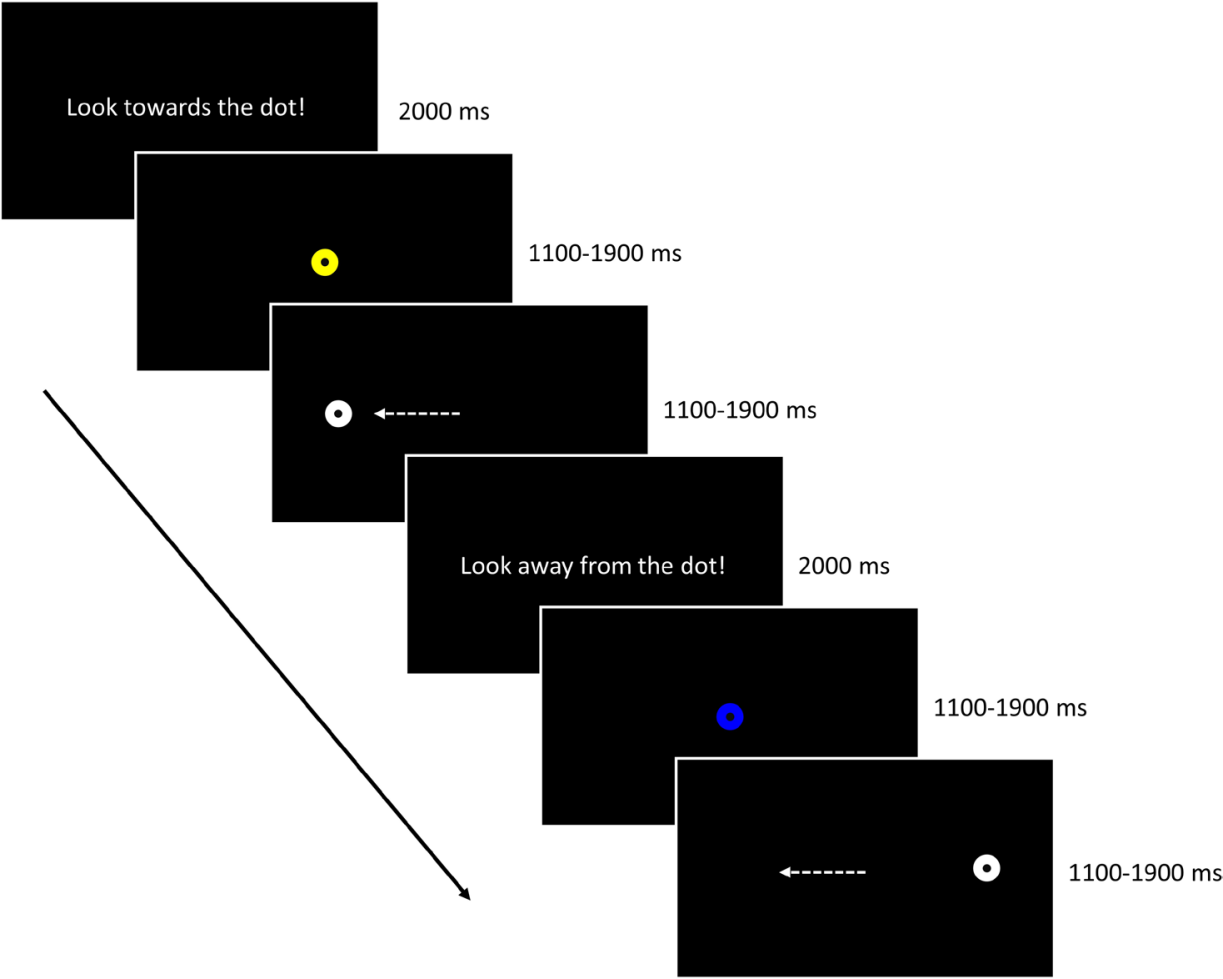
Schematic representation of the saccade task. *Note*. The upper three panels depict a prosaccade trial, the lower ones an antisaccade trial (stimuli not drawn to scale). Arrows indicate the required eye movement.

The saccade task was written in Experiment Builder (SR Research Ltd., Ontario, Canada, version 2.4.1) and presented on a 30‐in. monitor (medres, Cologne, Germany; 1600 × 1200 pixels). A block design was used, with five 30‐s blocks per condition (prosaccades, antisaccades, and fixation), as previously employed (Aichert et al. 2012). Each saccade block comprised 10 trials, requiring subjects to either follow a target moving from screen center to left or right (prosaccades) or to look at the mirror image location (antisaccades). Fixation blocks required participants to fixate a central target. For a schematic representation (see Figure 1).

### 2.4 Data Acquisition

Eye movements were recorded using an MR‐compatible video‐based combined pupil and corneal reflection eye tracker (EyeLink 1000, SR Research Ltd.) at 1000 Hz sampling rate. A five‐point horizontal‐vertical calibration was performed before the task. Blood oxygen level dependent (BOLD) data were acquired with a Magnetom 7 T Plus scanner (Siemens Healthineers, Erlangen, Germany) at DZNE, Bonn, using a single‐transmit/32‐receive (1Tx/32Rx) head coil (Nova Medical, Wilmington, USA). Functional data were acquired via an in‐house developed T2*‐weighted 3D‐echo‐planar imaging sequence (3D‐EPI; Stirnberg and Stöcker 2021) with following parameters: TR = 3000 ms, TE = 21 ms, flip angle = 15°, water excitation, matrix size = 216 × 216 × 128, voxel size = 1.0 × 1.0 × 1.0 mm, shot‐selective CAIPIRINHA acceleration scheme 4 × 2_y2_ (Hendriks et al. 2020), readout bandwidth = 1102 Hz/Px. For structural data, a T1‐weighted image was acquired using a custom magnetization‐prepared rapid acquisition gradient echo (MP‐RAGE) sequence (Brenner et al. 2014) with the following parameters: TR = 2500 ms, TE = 2.90 ms, flip angle = 7°, water excitation, matrix size = 364 × 428 × 256, voxel size = 0.6 × 0.6 × 0.6 mm, acceleration 2 × 1, readout bandwidth = 260 Hz/Px.

### 2.5 Oculomotor Data Analysis

Oculomotor data preprocessing was performed with EyeLink DataViewer software (SR Research Ltd., version 4.4.1) and R (version 4.4.2). Standard settings of EyeLink Data Viewer were used to detect saccades, with a minimum amplitude threshold (2°) to account for relatively noisy data. Trials with signal loss between saccade onset and offset were included in the analysis of latency and direction errors. Analysis of peak velocity and amplitude gain was based only on trials with full saccade signal. Amplitude gain was calculated as ratio of saccade amplitude to target amplitude. Trials with outlier values on peak velocity, amplitude gain and latency (based on 1.5 times the interquartile range) were excluded. Only data sets with at least seven valid trials per parameter were analyzed.

Linear mixed models were estimated separately for peak velocity, amplitude gain, latency and error rate, with drug (placebo, lorazepam) and condition (prosaccade, antisaccade) as fixed factors and subject as random factor, using the lme4 R package (version 1.1.36; Bates et al. 2015). Due to limitations in data quality, we deviated from the preregistration at this point and analyzed only those key variables using linear mixed models. Non‐normal residuals for error rate (*Q*–*Q* plots) were log(*x* + 1) transformed, resulting in a distribution closer to normal. *p*‐Values were obtained via Satterthwaite's degrees of freedom method using the lmerTest package (version 3.1.3; Kuznetsova et al. 2017). Comparisons of simpler with more complex models were performed via likelihood‐ratio tests (*χ* ^2^‐tests based on model deviances). Since no model benefited significantly from the inclusion of a drug × condition interaction term, only results for simpler models are reported (parameters of model comparisons are in Table S1). As a robustness check, we additionally performed Bayesian regression modeling using the brms R package (version 2.23.0; Bürkner 2017, 2018, 2021) to compare models with and without interaction between drug and condition. We used weakly informative priors for all model parameters, with normal (0, 1) priors for the intercept and fixed effects and exponential (1) priors for variance parameters. The model specifications were identical to those of the linear mixed‐effects models. Dependent variables were *z*‐standardized prior to analysis.

### 2.6 MRI Data Analysis

MRI data analyses were performed in SPM12 using MATLAB 2024b. For susceptibility distortion correction, voxel displacement maps were calculated using phase and magnitude images from field maps obtained with dual‐echo gradient‐echo sequences (TE1 = 3.06 ms, TE2 = 4.08 ms). Structural scans were segmented into gray matter, white matter, and cerebral spinal fluid (CSF). Preprocessing of functional images included realignment to the first image (six parameter rigid body spatial transformation), unwarping based on the voxel displacement map, coregistration with structural images, normalization into MNI space, and smoothing using a 6 mm full width at half maximum (FWHM) Gaussian kernel. Realignment parameters were used to assess head movements. No participant showed movements exceeding a previously defined threshold of 3 mm between consecutive volumes.

At first level, general linear models (GLM) were specified separately for each participant and session. Task conditions (prosaccades, antisaccades) were modeled with 30s boxcar functions and convolved with a canonical hemodynamic response function. Realignment parameters were added as nuisance regressors and a 128 s‐high‐pass filter was applied. Separate and combined regressors for pro‐ and antisaccades were estimated and compared against an implicit baseline. To investigate effects of drug and task condition, first‐level contrast images were entered into a second‐level full factorial analysis, with drug (placebo, lorazepam) and condition (prosaccade, antisaccade) as within‐subject factors. Results for effects of task condition were thresholded using a voxel‐level family‐wise error rate (FWE) corrected *p* < 0.05 with a minimum cluster size of 20 voxels. Results for effects of drug condition were thresholded using a voxel‐level threshold of *p* < 0.001 and a cluster‐size threshold of FWE‐corrected *p* < 0.05. The latter correction method was also applied to subsequent analyses of fMRI data, including functional connectivity and regression analyses. Anatomical and functional labels were determined using the SPM Anatomy Toolbox (Eickhoff et al. 2005) and the neuromorphometrics atlas in SPM for unlabeled regions. Since no interactions between drug and condition were found on behavioral or neural level, follow‐up analyses were performed for a combined saccade condition comprising pro‐ and antisaccades.

### 2.7 Functional Connectivity Analysis

We explored effects of drug and task condition on functional connectivity, using CONN 22.v2407 (Whitfield‐Gabrieli and Nieto‐Castanon 2012). Functional data were denoised using a standard denoising pipeline (Nieto‐Castanon 2020). For first‐level analyses, psychophysiological interaction (PPI) analyses were used to study the changes in functional connectivity across conditions. Seed‐to‐voxel generalized PPI (gPPI) analyses were performed with seed regions including 8 regions of interest (ROIs; bilateral V1, posterior IPS, anterior IPS, medial FEF). These regions are essential to the saccadic network (Ettinger et al. 2008; Jamadar et al. 2013; Milner and Goodale 1992; Vossel et al. 2014). Functional connectivity changes across conditions were characterized by Fisher‐transformed semipartial correlation coefficients of PPI terms in each model. Second‐level analyses were performed, conducting paired *t*‐tests for lorazepam vs. placebo to assess drug‐dependent connectivity during the combined saccade condition. Finally, we calculated change scores (placebo—lorazepam) across conditions for connectivity clusters with significant effects and behavioral and subjective variables showing significant drug effects. Change scores were then correlated to investigate associations of connectivity results with behavioral and subjective measures.

### 2.8 Individual Differences

To characterize neural correlates of individual differences in oculomotor responses to lorazepam (Ettinger et al. 2018), regression analyses were performed to identify areas where greater behavioral performance decline from placebo to lorazepam was associated with greater BOLD changes. Difference images (placebo–lorazepam) were calculated using first‐level contrast images (combined pro‐ and antisaccades > baseline) and entered into second‐level multiple regression analyses. Corresponding behavioral change scores were entered as covariates in separate analyses. Only subjects with oculomotor and fMRI data from both conditions were included (*N* = 27).

Additional regression analyses on BOLD data were performed with plasma level and VAS subscales showing significant drug effects as covariates (see Supporting Information Results).

### 2.9 Associations With GABA_A_ Receptor Density

To explore molecular explanations for lorazepam‐induced BOLD effects, in a non‐preregistered analysis we examined associations with GABA_A_ receptor density using a publicly available atlas (Nørgaard et al. 2021). Therefore, we first compared GABA_A_ receptor densities (pmol/mL) between regions with significant drug effects and those without. Because receptor density in cortical areas is higher than in subcortical and brainstem structures (Nørgaard et al. 2021), we further compared receptor densities between cortical regions with and without significant drug effects. Finally, we ran voxel‐wise correlations between GABA_A_ receptor density and the magnitude of lorazepam effects using the placebo > lorazepam contrast.

## 3 Results

### 3.1 Participants

**FIGURE 2:**
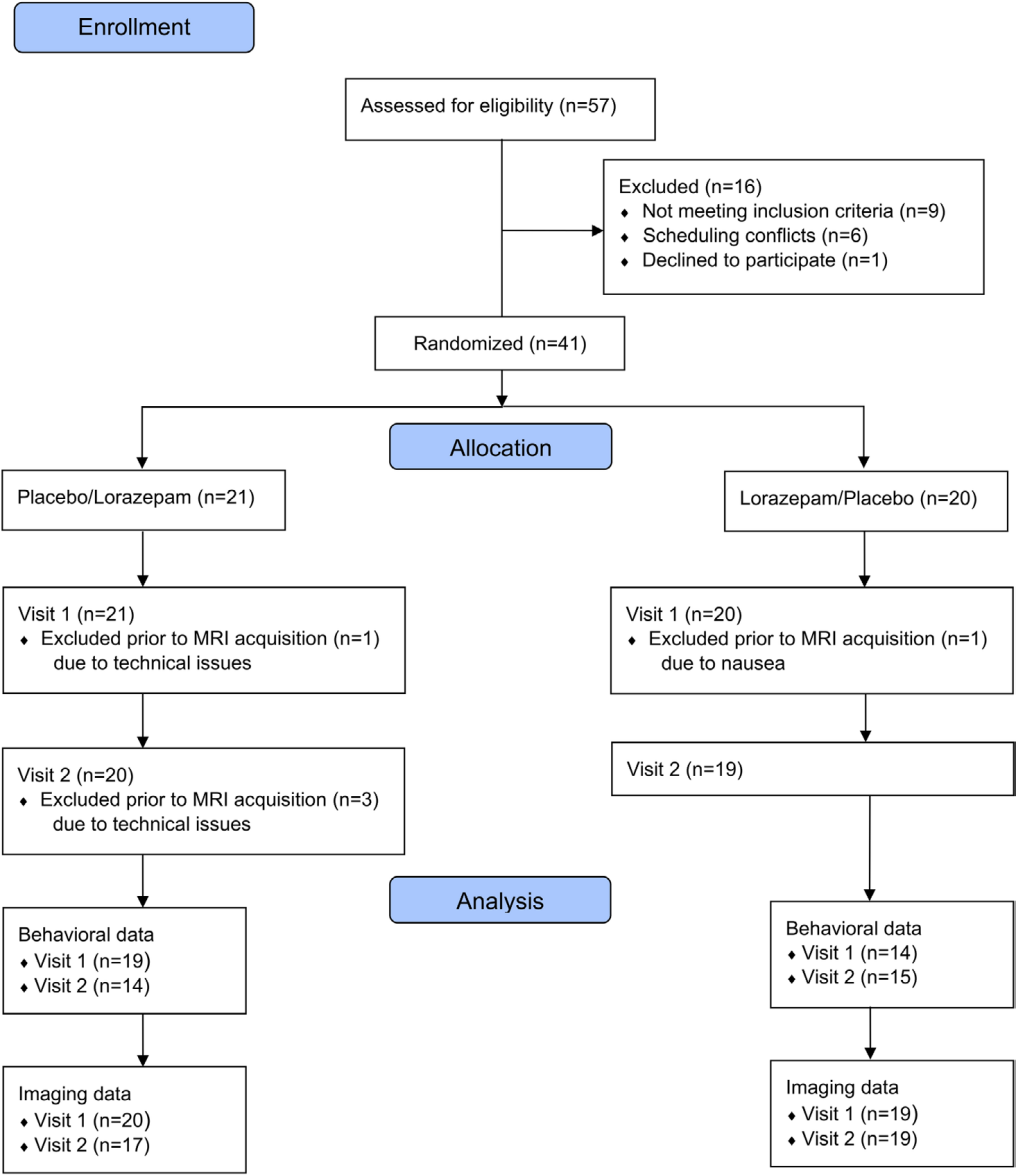
Consort diagram. *Note*. The diagram illustrates participant exclusion at different stages of the study.

The final sample included 39 participants (28 female, 11 male) with at least one complete MRI session. Thirty‐six participants completed both sessions. Mean age of full sample was 25.10 years (SD = 4.30). Three participants were excluded from MRI but included in oculomotor data analysis. Results did not change without their inclusion. A full overview is in Figure 2.

### 3.2 Subjective Ratings, Vital Signs and Plasma Levels

Under lorazepam, participants felt significantly less alert and content (both *p* < 0.001), showed a significantly higher pulse (*p* < 0.01), and had significantly higher lorazepam plasma levels (*p* < 0.001). No differences were found for calmness or blood pressure (all *p* > 0.24). Further details are in the Supplementary Results.

### 3.3 Behavioral Data

Descriptive results are in Table 1. Detailed model parameters for linear mixed‐effect models are in Table S2. Across all models, fixed effect of drug was significant for peak velocity (*b* = −24.33, SE = 5.19, 95% CI [−34.51, −14.15], *t*(71.68) = −4.69, *p* < 0.001) and latency (*b* = 10.18, SE = 3.69, 95% CI [2.95, 17.40], *t*(81.43) = 2.76, *p* = 0.007), indicating decreased peak velocity and increased latency under lorazepam, as expected. Fixed effect of condition was significant for peak velocity (*b* = −20.09, SE = 5.20, 95% CI [−30.28, −9.91], *t*(71.48) = −3.87, *p* < 0.001), latency (*b* = 68.91, SE = 3.62, 95% CI [61.82, 76.00], *t*(78.55) = 19.05, *p* < 0.001) and error rate (*b* = 2.32, SE = 0.15, 95% CI [2.02, 2.62], *t*(86.29) = 14.99, *p* < 0.001), indicating decreased peak velocity and increased latency and error rate for antisaccades. Results for peak velocity did not change after correction for amplitude (see Supplementary Results).

**TABLE 1:** Descriptive statistics of oculomotor data.

	Placebo		Lorazepam	
	Prosaccades	Antisaccades	Prosaccades	Antisaccades
Peak velocity (°/s)	284.36 (42.85)	267.66 (40.08)	259.89 (42.36)	245.21 (41.11)
	*n* = 34	*n* = 26	*n* = 27	*n* = 18
Amplitude (°)	6.08 (0.62)	6.29 (0.72)	6.05 (0.53)	6.04 (0.91)
	*n* = 34	*n* = 26	*n* = 27	*n* = 18
Amplitude gain (ratio)	0.94 (0.08)	0.95 (0.11)	0.92 (0.07)	0.92 (0.14)
	*n* = 34	*n* = 26	*n* = 27	*n* = 18
Latency (ms)	224.06 (20.90)	292.61 (27.63)	231.28 (22.47)	302.51 (28.75)
	*n* = 34	*n* = 28	*n* = 27	*n* = 22
Error rate (%)	0.75 (2.52)	19.91 (23.34)	2.04 (7.05)	24.57 (22.97)
	*n* = 34	*n* = 33	*n* = 27	*n* = 24

*Note:* Values refer to means for correct saccades (standard deviations). Error rate was calculated based on all analyzable trials. Sample sizes differ across parameters, drug and condition due to limitations in data quality and the application of parameter‐specific filtering (see Methods).

Bayesian regression modeling supported the null findings regarding the interaction between drug and condition. Model comparison using leave‐one‐out cross‐validation indicated only minor differences in model fit between models with and without interaction term for peak velocity (difference in expected log predictive density (ELPD): *Δ* = −0.9; SE = 0.8), amplitude gain (*Δ* = −0.9, SE = 0.5), latency (*Δ* = −1.4, SE = 0.7), or error rate (*Δ* = −0.2, SE = 1.4). For peak velocity, amplitude gain and latency, models without the interaction term showed slightly better predictive performance, whereas for error rate, the model with the interaction term showed slightly better predictive performance. However, the difference in ELPD for error rate cannot be interpreted as evidence in favor of the model with the interaction term, as it remains small compared to its standard error (Vehtari et al. 2017). It should be noted that ELPD values can show minor numerical variations across repeated computations.

### 3.4 Imaging Data

#### 3.4.1 Whole‐Brain Analysis

Both the main effect of saccade condition and direct contrasts between pro‐ and antisaccades against each other revealed activation within a well‐known, saccadic fronto‐parietal‐occipital network, validating the present paradigm (Tables S3–S5 and Figures S1–S3).

**FIGURE 3:**
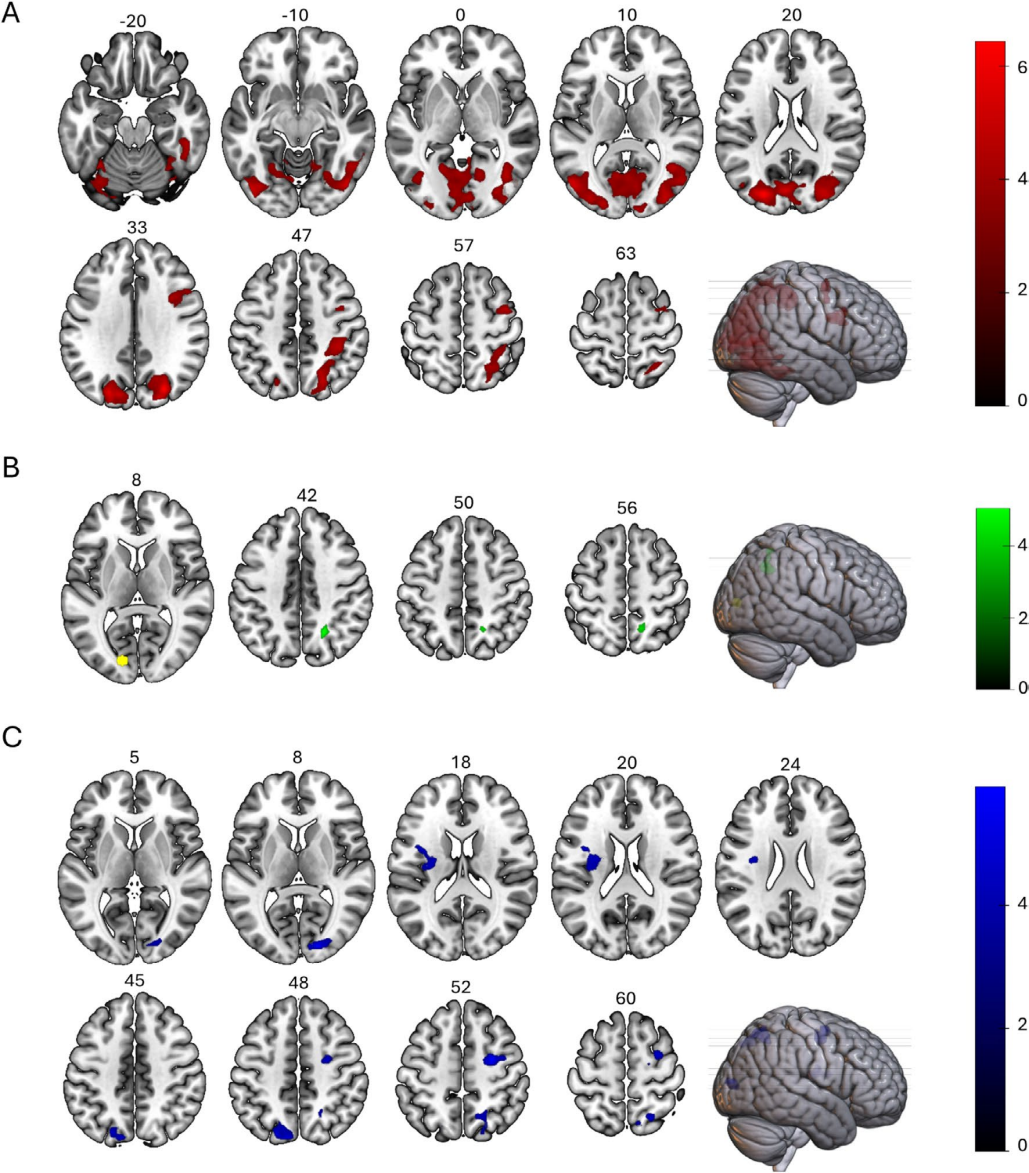
Lorazepam effects on brain activity. *Note*. (A) Reduced activation during pro‐and antisaccade performance under lorazepam compared to placebo. (B) Cluster with positive connectivity (green) to seed voxels in left V1 (yellow) under lorazepam compared to placebo. (C) Individual differences in the extent of reduced activation during pro‐and antisaccade performance under lorazepam compared to placebo, predicted by individual decline in saccadic peak velocity.

**FIGURE 4:**
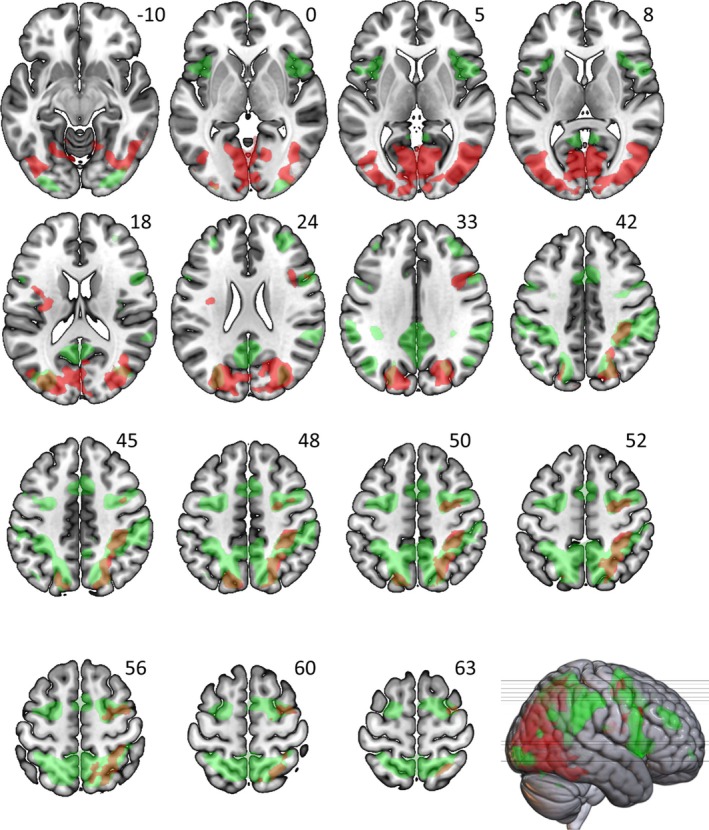
Overlap between different expressions of the drug effect and the main effect of saccade condition. *Note*. Voxel‐level threshold for the main effect of saccade condition (including pro‐ and antisaccade condition; green) was set to *p* < 0.05, FWE‐corrected; cluster threshold was set to 20 voxels. Voxel‐level threshold for drug effects (red; including the whole‐brain analysis, functional connectivity analysis and regression analysis) was set to *p* < 0.001, uncorrected; cluster‐level threshold was set to *p* < 0.05, FWE‐corrected. All clusters were converted to binary masks to highlight overlaps between regions.

Across saccade conditions, reduced activation with lorazepam than placebo was found in (1) a large parieto‐occipito‐temporal cluster, encompassing visual areas (V1–V6), IPS and superior parietal lobule (SPL) and extending into precuneus, right middle and inferior temporal gyri, right postcentral gyrus and cerebellum. Additionally, two smaller, right‐hemispheric clusters showed reduced activation under lorazepam in (2) precentral gyrus, middle and inferior frontal gyri, and (3) medial and lateral FEF (Table 2, Figure 3A). These clusters (87,807 voxels) overlapped partially with the pro‐/antisaccade network (144,469 voxels; 18,985 voxels overlap; Figure 4).

**TABLE 2:** Blood oxygen level dependent (BOLD) activation for placebo vs. lorazepam across saccade conditions.

Anatomical label (functional label)	Cluster size	*t*	MNI coordinates		
			*x*	*y*	*z*
R posterior intraparietal sulcus	84,310	6.49	28	−76	32
L posterior intraparietal sulcus		6.46	−24	−84	19
L inferior lateral occipital cortex		5.81	−45	−77	−11
L cuneal cortex (V3d)		5.65	−5	−81	18
R inferior temporal gyrus		5.53	49	−60	−16
R inferior lateral occipital cortex		5.39	47	−71	−12
R precentral gyrus	2246	4.67	39	9	28
R middle frontal gyrus		3.91	49	15	34
R inferior frontal gyrus		3.51	53	13	24
R precentral gyrus (lateral FEF)	1251	4.56	40	−2	57
R middle frontal gyrus (medial FEF)		3.31	30	−3	63

*Note:* Voxel‐level threshold was set to *p* < 0.001, uncorrected. Cluster‐level threshold was set to *p* < 0.05, FWE‐corrected. V3d = dorsal V3. Lateral FEF = lateral frontal eye fields. Medial FEF = medial frontal eye fields. For each defined region, only the peak with the highest *t*‐value is presented; the complete set of peaks can be found in Supporting Information (Table S6).

No significant activation was found for the reverse contrast (lorazepam > placebo). Again, there was no significant interaction between drug and condition, shifting the focus of subsequent analyses to more detailed exploration of condition‐independent lorazepam effects.

#### 3.4.2 Functional Connectivity Analysis

**FIGURE 5:**
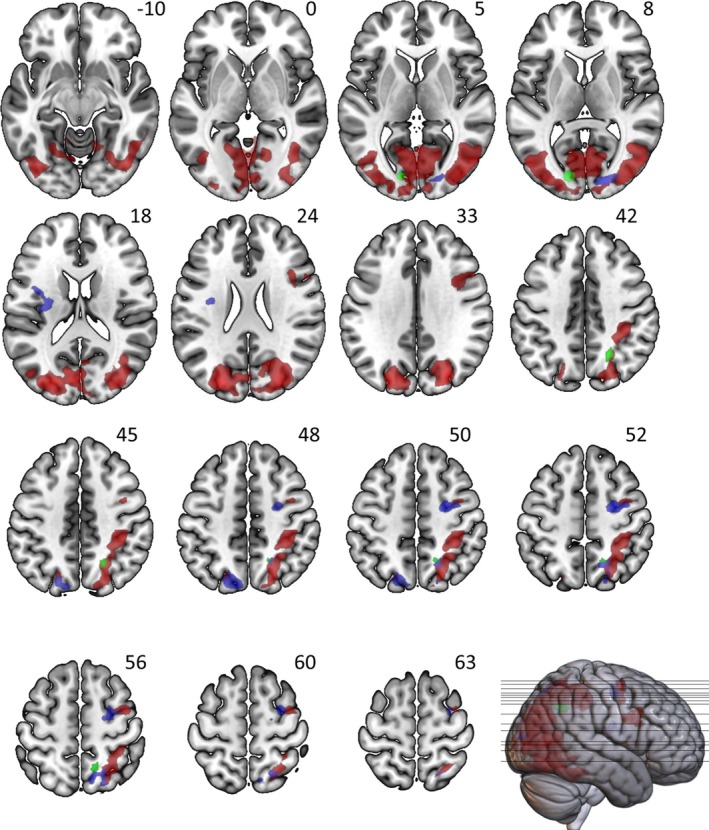
Overlap between different expressions of the drug effect. *Note*. The BZD‐modulated network is shown in red. Regions showing positive connectivity under lorazepam compared to placebo are shown in green. Regions with individual differences in the extent of reduced activation during pro‐and antisaccade performance under lorazepam compared to placebo, predicted by individual decline in saccadic peak velocity, are shown in blue.

Connectivity differed between drug conditions only for the left V1 seed, with a cluster comprising right SPL, posterior IPS, and precuneus (Table 3 and Figure 3B). Connectivity was positive under lorazepam and negative under placebo, indicating that under lorazepam, higher activation in the seed was associated with higher activation in the cluster, whereas under placebo, higher seed activation was associated with lower activation in the cluster. Seed and cluster (1560 voxels) partially overlapped with the pro‐/antisaccade network (580 voxels overlap; Figure 4) and the general drug effect (337 voxels overlap; Figure 5). Changes in connectivity were not associated with changes in behavioral and subjective measures showing significant drug effects (all *p* ≥ 0.084).

**TABLE 3:** Clusters with significant connectivity differences to the respective seed region between placebo and lorazepam.

Anatomical label (functional label)	Cluster size	*t*	MNI coordinates		
			*x*	*y*	*z*
Seed region: left V1					
R lateral occipital cortex, superior division	123	5.18	22	−58	42
R superior parietal lobule (IPS)		4.57	18	−56	50
R precuneus (SPL)		4.26	14	−54	56

*Note:* For left V1, connectivity to target cluster was negative for placebo and positive for lorazepam (*t*(35) = 6.21, *p* < 0.001). Voxel‐level threshold was set to *p* < 0.001, uncorrected. Cluster‐level threshold was set to *p* < 0.05, FWE‐corrected.

#### 3.4.3 Individual Differences

In voxel‐wise regression analyses, change scores for peak velocity were significantly positively associated with changes in neural activity. Stronger decline in peak velocity with lorazepam was associated with greater reduction in activation during lorazepam in five clusters, including right superior and middle frontal gyri, precentral gyrus (medial FEF), bilateral IPS and SPL, right occipital pole (V1–V3) as well as left insular and central opercular cortex (Table 4 and Figure 3C). These clusters (6971 voxels) showed large overlap with the pro‐/antisaccade network (4257 voxels overlap; Figure 4), but relatively minor overlap with the general drug effect (1413 voxels overlap; Figure 5).

**TABLE 4:** Results from regression analyses on first‐level difference images (placebo‐lorazepam) for combined pro‐ and antisaccades > baseline, with behavioral change scores for peak velocity (placebo‐lorazepam) as predictor (positive association).

Anatomical label (functional label)	Cluster size	*t*	MNI coordinates		
			*x*	*y*	*z*
R occipital pole (V1)	1409	5.98	18	−90	8
R occipital pole (V3d)		5.33	23	−93	12
R precentral gyrus (medial FEF)	1737	5.98	27	−7	52
R superior frontal gyrus (medial FEF)		4.67	29	−2	60
L lateral occipital cortex, superior division (IPS)	1087	5.23	−11	−79	47
L lateral occipital cortex, superior division (SPL)		3.58	−18	−66	49
NA (L cerebral white matter)	1193	4.88	−39	1	20
R lateral occipital cortex, superior division (SPL)	1545	4.74	23	−61	59
R lateral occipital cortex, superior division (area 7A, SPL)		4.59	14	−60	53
R lateral occipital cortex, superior division (IPS)		4.09	20	−59	51
R superior parietal lobule (IPS)		3.89	22	−56	53

*Note: N* = 27. Voxel‐level threshold was set to *p* < 0.001, uncorrected. Cluster‐level threshold was set to *p* < 0.05, FWE‐corrected. V3d = dorsal V3. Medial FEF = medial frontal eye fields. IPS = intraparietal sulcus. SPL = superior parietal lobule. For each defined region, only the peak with the highest *t*‐value is presented; the complete set of peaks can be found in the Supplement (Table S7).

There were no significant results when using latency change score as a predictor.

#### 3.4.4 Associations With GABA_A_ Receptor Density

Drawing upon the atlas by Nørgaard et al. (2021), we found that GABA_A_ receptor density was significantly higher in regions with significant drug effects (*M* = 636.56, SD = 356.75, Figure S4), compared to both remaining brain areas (*M* = 104.60, SD = 241.16; *t*(88 796.53) = 440.61, *p* < 0.001, *d* = 2.19) and remaining cortical areas (*M* = 456.84, SD = 338.21; *t*(100 421) = 144.34, *p* < 0.001, *d* = 0.53).

Further, there were significant voxel‐wise correlations between magnitude of lorazepam effects on BOLD during saccades and GABA_A_ receptor density, both within regions with significant drug effects (*r*(87, 805) = 0.019, 95% CI [0.013, 0.026] *p* < 0.001) and the whole brain (*r*(7, 109, 135) = 0.231, 95% CI [0.231, 0.232] *p* < 0.001).

Together, results indicate that reductions in BOLD with lorazepam were stronger in voxels with higher GABA_A_ receptor densities.

## 4 Discussion

Using a double‐blind, placebo‐controlled within‐subjects design, this study replicated the observation of increased latency and decreased peak velocity after lorazepam administration (Bey et al. 2021; Ettinger et al. 2018; Faßbender et al. 2021; Green et al. 2000; Green and King 1998; McCartan et al. 2001) across pro‐ and antisaccade conditions. For the first time, neural correlates of these effects were investigated, revealing decreased activation with lorazepam in several regions of the saccadic network, including temporo‐parieto‐occipital cortex and right FEF. Importantly, when compared to unaffected regions, these areas showed higher GABA_A_ receptor densities, and densities were correlated with the strength of lorazepam effects at voxel‐level. Task‐dependent connectivity changes between drug conditions were observed for the left V1 seed. Beyond these group‐level drug effects, associations between individual reductions in saccadic peak velocity could be tracked to reduced neural activations in saccade‐related regions, including visual areas and medial FEF.

### 4.1 GABAergic Modulation of the Saccadic Network

As expected (Bey et al. 2021; Ettinger et al. 2018; Faßbender et al. 2021; McCartan et al. 2001), the pro‐/antisaccade paradigm was suitable to characterize lorazepam effects not only on behavioral but also neural level. Lorazepam effects were not specific to task condition, as increased latency and reduced peak velocity were observed across anti‐ and prosaccades, and effects on BOLD occurred within the general visuo‐saccadic network rather than in specific inhibitory control regions. This lack of an interaction effect was also supported by Bayesian regression modeling of the oculomotor data. Behavioral results therefore partly differ from previous findings of BZD‐induced impairments in inhibitory control and antisaccade performance (Faßbender et al. 2021; Sarkar et al. 2020). However, the alignment of behavioral and neural results as well as the effects on regions associated with both pro‐ and antisaccades (Brown et al. 2006; Ettinger et al. 2008; Jamadar et al. 2013) suggests that similar GABAergic mechanisms underlie automatic and voluntary saccades. Accordingly, regions that earlier studies have reported to be specifically activated during antisaccades, like DLPFC or ACC (Jamadar et al. 2013; McDowell et al. 2008), did not show any lorazepam effects, neither at group level nor in terms of individual differences.

Compared to studies examining excitatory or modulatory neurotransmitter systems, reporting modulation of prefrontal regions during cognitive control (Driesen et al. 2013; Honey et al. 2004; Mehta et al. 2000), GABAergic modulation of saccade performance thus primarily involved sensorimotor and oculomotor regions (Logue and Gould 2014). Further analysis showed that GABA_A_ receptor density was higher in affected than unaffected regions. However, although prefrontal regions also exhibit comparatively high receptor densities (Nørgaard et al. 2021), they were not affected by lorazepam. This further supports our conclusion of a lack of specific drug effects on inhibitory control. Thus, GABAergic modulation occurs particularly in areas associated with more fundamental saccadic and visuomotor processes. The significant correlation between the strength of the lorazepam effect and GABA_A_ receptor density further supports a GABAergic drug mechanism rather than performance‐related confounds (Connolly et al. 2005; Pierce and Mcdowell 2016).

The sensitivity of the (anti‐)saccade task to BZD effects is further supported by comparison with other cognitive domains. Specifically, in an exploratory analysis reported in the Supplement, we chose an intertemporal choice paradigm (Kirby 2009) as a representative comparison task, as there is no evidence of BZD effects on behavioral measures in this task (Sarmiento et al. 2023). We performed similar analysis steps for GABA_A_ receptor densities in intertemporal choice task‐relevant brain areas (Bartra et al. 2013). While GABA_A_ receptor density in those areas was significantly higher compared to remaining brain areas, it was significantly lower than in our saccadic network (see Supporting Information Methods and Results). Taken together, these findings support the use of (anti‐)saccade performance and accompanying neural measures as highly sensitive markers of BZD activity.

### 4.2 Region‐Specific GABA Modulations Within the Saccadic Network

One of the most prominent regions within the identified BZD‐sensitive network was the IPS, an area repeatedly associated with pro‐ and antisaccade performance (Berman et al. 1999; Brown et al. 2006; Ettinger et al. 2008; Jamadar et al. 2013). Interestingly, lorazepam‐induced BOLD reductions affected bilateral posterior IPS, but only right anterior IPS, a region previously associated with stronger activation during antisaccade generation compared to saccade inhibition and prosaccade generation (Ettinger et al. 2008). This again confirms GABAergic modulation of execution‐related regions, but also points to an antisaccade‐specific lorazepam effect. However, this selectivity for right anterior IPS does not undermine our general interpretation of a rather condition‐unspecific effect on the saccadic network, since previous studies reported neural modulation by lorazepam without significant changes in behavior (Arce et al. 2006; Paulus et al. 2005; Schunck et al. 2010; Walter et al. 2016). Thus, while general effects on the saccadic network might be stronger and evident at both neural and behavioral levels, antisaccade‐specific effects appear to be reflected only in neural modulations, with lesser or nonspecific manifestations at the behavioral level. We therefore conclude that BOLD signal may be a more sensitive marker for lorazepam activity than behavior, a pattern that was previously found for other psychoactive drugs (Costa et al. 2013; Hershey et al. 2004; Kasparbauer et al. 2019; Steffens et al. 2018).

Right posterior IPS further showed positive connectivity from left V1 under lorazepam, while negative connectivity occurred under placebo. Since both regions partially overlapped with the BZD‐affected network (Figure 5), this finding points to an altered neuronal strategy across affected regions. Given that connectivity between V1 and IPS can be modified by attentional processes (Griffis et al. 2015), synchronized activation under lorazepam could point to stronger reliance on stimulus‐driven signals, whereas signals from higher‐level cortical regions contribute more strongly to performance under placebo. Considering the overall attenuated activation under lorazepam and the overlap of the connectivity pattern with the affected network, this finding may also reflect general dampening rather than more differentiated coupling under placebo.

Another prominent region affected by lorazepam was right FEF. While lorazepam‐related reductions were found for more lateral portions of FEF in the whole‐brain analysis at group level, an association with a decrease in saccadic peak velocity was found for medial FEF on an individual level (Figure 5). Our finding that other predictors, including behavioral, subjective, and pharmacokinetic parameters, could not explain changes in BOLD signal, emphasizes the specificity of this result. In line with previous studies reporting functional differences between lateral and medial FEF (Cieslik et al. 2016), we assume a more general effect of lorazepam in comparatively lateral portions of FEF, while medial FEF seems to be specifically related to saccadic peak velocity. Despite our findings of increased saccadic latencies under lorazepam, no associations with BOLD signal change were detected. While a more global reduction in the affected saccadic network possibly accounts for this effect, we could not detect it using our voxel‐based individual differences approach.

Another notable observation regarding neural correlates of decreased saccadic peak velocity was its exclusive association with cortical areas on an individual level. Aside from some involvement of the cerebellum, subcortical regions were unaffected by BZD. These findings are somewhat surprising since saccadic peak velocity is typically associated with, among others, burst neuron activity in the brainstem (Fuchs et al. 1985; Sparks 2002). Although GABA_A_ receptor densities are low in this region (Nørgaard et al. 2021), this does not rule out the brainstem as an important area for saccadic peak velocity. However, it does suggest that GABAergic modulation of saccades occurs more prominently in cortical areas. Furthermore, these areas show considerable overlap with the general pro‐/antisaccade network (Figure 4), suggesting that GABAergic modulation affects functionally relevant regions.

A limitation of this study is that the block design did not allow the investigation of trial‐specific neural activation or its relationship to trial‐by‐trial variability in behavioral measures. For example, previous studies reported differences in neural activation between correct and error antisaccade trials (Ford et al. 2005; Polli et al. 2005). Therefore, we cannot rule out that averaging across blocks may have reduced the sensitivity of our study to detect drug effects specific to directionally correct antisaccades (i.e., a drug × condition interaction). A further limitation is that participants showed increased eyelid closure under lorazepam, which could represent an artifact in the analysis. However, we calculated change scores for eye‐tracking signal loss as proxy for eyelid closure and entered this variable into a regression model as for other variables (see Supporting Information Methods and Results). No reductions in BOLD signal were explained by increased signal loss under lorazepam, therefore this limitation may be of less concern. Finally, it should be noted that although correction for multiple comparisons was applied within each second‐level analysis, it cannot be ruled out that the overall type I error rate may have been inflated across the different analyses that were carried out.

## 5 Conclusion

To conclude, we demonstrated for the first time the neural correlates of lorazepam effects on the saccadic network. Performance declines in saccadic peak velocity and latency were accompanied by decreased neural activation in key oculomotor regions, indicating GABAergic modulation of a more general saccade‐related network rather than of specific components for inhibitory control processes. This finding was supported by evidence of increased GABA_A_ receptor density in the affected network and by associations with individual decline in saccadic peak velocity. Future studies therefore may rely on BOLD signal as a sensitive marker for BZD activity during saccadic eye movements.

## Author Contributions

P.Q. and U.E. made substantial contributions to the conceptualization and design of the study, data acquisition, data analysis, and the writing of the original draft of the manuscript. P.‐M.S., K.F., and P.M.B. were involved in the conception and design of the study. S.M., B.A., M.G., L.v.d.E., and R.L. provided support during data acquisition. B.S.‐W. and R.D.‐P. oversaw the blood sample analyses and contributed laboratory resources. R.S. and T.S. were responsible for the development of the MRI sequences.

## Funding

The authors have nothing to report. During the study period, Pia‐Magdalena Schmidt was financially supported by doctoral scholarships from the Claussen‐Simon Foundation (Hamburg, Germany) and the Cusanuswerk e.V. (Bonn, Germany).

## Conflicts of Interest

The authors declare no conflicts of interest.

## Supporting information


**Table S1:** Model evaluation metrics for models with and without interaction terms.
**Table S2:** Linear mixed models for oculomotor data.
**Table S3:** Blood oxygen level dependent (BOLD) activation for the main effect of condition (pro‐ and antisaccades).
**Table S4:** Blood oxygen level dependent (BOLD) activation for the antisaccade > prosaccade contrast.
**Table S5:** Blood oxygen level dependent (BOLD) activation for the prosaccade > antisaccade contrast.
**Table S6:** Blood oxygen level dependent (BOLD) activation for placebo vs. lorazepam across saccade conditions (extended version of Table 2).
**Table S7:** Results from regression analyses on first‐level difference images (placebo‐lorazepam) for combined pro‐ and antisaccades > baseline (extended version of Table 4).
**Figure S1:** Brain activation for the main effect of saccade condition.
**Figure S2:** Brain activation for the antisaccade > prosaccade contrast.
**Figure S3:** Brain activation for the prosaccade > antisaccade contrast.
**Figure S4:** GABA_A_ receptor density within the BZD‐modulated network.

## Data Availability

The behavioral data supporting the findings of this study are available on the Open Science Framework (OSF) at https://osf.io/apsyv/overview?view_only=885cc4b78a404e71986b47b90d889320. Due to ethical restrictions and data protection regulations, MRI data are not publicly available but are available from the corresponding author upon reasonable request.

## References

[hbm70598-bib-0001] Aichert, D. S. , S. C. R. Williams , H. J. Möller , V. Kumari , and U. Ettinger . 2012. “Functional Neural Correlates of Psychometric Schizotypy: An fMRI Study of Antisaccades.” Psychophysiology 49: 345–356. 22091533 10.1111/j.1469-8986.2011.01306.x

[hbm70598-bib-0002] Arce, E. , D. A. Miller , J. S. Feinstein , M. B. Stein , and M. P. Paulus . 2006. “Lorazepam Dose‐Dependently Decreases Risk‐Taking Related Activation in Limbic Areas.” Psychopharmacology 189: 105–116. 17016713 10.1007/s00213-006-0519-8 PMC2839080

[hbm70598-bib-0003] Baldwin, D. S. , K. Aitchison , A. Bateson , et al. 2013. “Benzodiazepines: Risks and Benefits. A Reconsideration.” Journal of Psychopharmacology 27: 967–971. 24067791 10.1177/0269881113503509

[hbm70598-bib-0004] Bartra, O. , J. T. McGuire , and J. W. Kable . 2013. “The Valuation System: A Coordinate‐Based Meta‐Analysis of BOLD fMRI Experiments Examining Neural Correlates of Subjective Value.” NeuroImage 76: 412–427. 23507394 10.1016/j.neuroimage.2013.02.063 PMC3756836

[hbm70598-bib-0005] Bates, D. , M. Mächler , B. M. Bolker , and S. C. Walker . 2015. “Fitting Linear Mixed‐Effects Models Using LME4.” Journal of Statistical Software 67: 1–48.

[hbm70598-bib-0006] Baumert, P. M. , K. Faßbender , M. W. M. Wintergerst , et al. 2024. “Effects of Lorazepam on Saccadic Eye Movements—Evidence From Prosaccade and Free Viewing Tasks.” Psychopharmacology (Berlin) 242: 271–284. 39225714 10.1007/s00213-024-06672-z PMC11775061

[hbm70598-bib-0007] Berman, R. A. , C. L. Colby , C. R. Genovese , et al. 1999. “Cortical Networks Subserving Pursuit and Saccadic Eye Movements in Humans: An FMRI Study.” Human Brain Mapping 8: 209–225. 10619415 10.1002/(SICI)1097-0193(1999)8:4<209::AID-HBM5>3.0.CO;2-0 PMC6873313

[hbm70598-bib-0008] Bey, K. , J. V. Lippold , B. Aslan , R. Hurlemann , and U. Ettinger . 2021. “Effects of Lorazepam on Prosaccades and Saccadic Adaptation.” Journal of Psychopharmacology 35: 91–99. 33274663 10.1177/0269881120972424

[hbm70598-bib-0009] Bond, A. , and M. Lader . 1974. “The Use of Analogue Scales in Rating Subjective Feelings.” British Journal of Medical Psychology 47: 211–218.

[hbm70598-bib-0010] Boucart, M. , N. Waucquier , G. A. Michael , and C. Libersa . 2007. “Diazepam Impairs Temporal Dynamics of Visual Attention.” Experimental and Clinical Psychopharmacology 15: 115–122. 17295590 10.1037/1064-1297.15.1.115

[hbm70598-bib-0011] Brenner, D. , R. Stirnberg , E. D. Pracht , and T. Stöcker . 2014. “Two‐Dimensional Accelerated MP‐RAGE Imaging With Flexible Linear Reordering.” Magma 27: 455–462. 24510154 10.1007/s10334-014-0430-y

[hbm70598-bib-0012] Brown, M. R. G. , H. C. Goltz , T. Vilis , K. A. Ford , and S. Everling . 2006. “Inhibition and Generation of Saccades: Rapid Event‐Related fMRI of Prosaccades, Antisaccades, and Nogo Trials.” NeuroImage 33: 644–659. 16949303 10.1016/j.neuroimage.2006.07.002

[hbm70598-bib-0013] Bürkner, P. C. 2017. “Brms: An R Package for Bayesian Multilevel Models Using Stan.” Journal of Statistical Software 80: 1–28.

[hbm70598-bib-0014] Bürkner, P. C. 2018. “Advanced Bayesian Multilevel Modeling With the R Package Brms.” R Journal 10: 395–411.

[hbm70598-bib-0015] Bürkner, P. C. 2021. “Bayesian Item Response Modeling in R With BRMS and Stan.” Journal of Statistical Software 100: 1–54.

[hbm70598-bib-0016] Chen, X. , G. Jacobs , M. L. De Kam , et al. 2015. “AZD6280, a Novel Partial γ‐Aminobutyric Acid a Receptor Modulator, Demonstrates a Pharmacodynamically Selective Effect Profile in Healthy Male Volunteers.” Journal of Clinical Psychopharmacology 35: 22–33. 25493397 10.1097/JCP.0000000000000251

[hbm70598-bib-0017] Cieslik, E. C. , I. Seidler , A. R. Laird , P. T. Fox , and S. B. Eickhoff . 2016. “Different Involvement of Subregions Within Dorsal Premotor and Medial Frontal Cortex for Pro‐ and Antisaccades.” Neuroscience and Biobehavioral Reviews 68: 256–269. 27211526 10.1016/j.neubiorev.2016.05.012 PMC5003685

[hbm70598-bib-0018] Cloos, J. M. , and V. Ferreira . 2009. “Current Use of Benzodiazepines in Anxiety Disorders.” Current Opinion in Psychiatry 22: 90–95. 19122540 10.1097/YCO.0b013e32831a473d

[hbm70598-bib-0019] Connolly, J. D. , M. A. Goodale , R. S. Menon , and D. P. Munoz . 2002. “Human fMRI Evidence for the Neural Correlates of Preparatory Set.” Nature Neuroscience 5: 1345–1352. 12411958 10.1038/nn969

[hbm70598-bib-0020] Connolly, J. D. , M. A. Goodale , H. C. Goltz , and D. P. Munoz . 2005. “fMRI Activation in the Human Frontal Eye Field Is Correlated With Saccadic Reaction Time.” Journal of Neurophysiology 94: 605–611. 15590732 10.1152/jn.00830.2004

[hbm70598-bib-0021] Costa, A. , M. Riedel , O. Pogarell , et al. 2013. “Methylphenidate Effects on Neural Activity During Response Inhibition in Healthy Humans.” Cerebral Cortex 23: 1179–1189. 22581848 10.1093/cercor/bhs107

[hbm70598-bib-0022] Crowe, S. F. , and E. K. Stranks . 2018. “The Residual Medium and Long‐Term Cognitive Effects of Benzodiazepine Use: An Updated Meta‐Analysis.” Archives of Clinical Neuropsychology 33: 901–911. 29244060 10.1093/arclin/acx120

[hbm70598-bib-0023] De Haas, S. L. , S. J. De Visser , J. P. Van Der Post , et al. 2007. “Pharmacodynamic and Pharmacokinetic Effects of TPA023, a GABAA α2,3 Subtype‐Selective Agonist, Compared to Lorazepam and Placebo in Healthy Volunteers.” Journal of Psychopharmacology 21: 374–383. 17092968 10.1177/0269881106072343

[hbm70598-bib-0024] De Visser, S. J. , J. P. Van Der Post , P. P. De Waal , F. Cornet , A. F. Cohen , and J. M. A. Van Gerven . 2003. “Biomarkers for the Effects of Benzodiazepines in Healthy Volunteers.” British Journal of Clinical Pharmacology 55: 39–50. 12534639 10.1046/j.1365-2125.2002.t01-10-01714.x PMC1884188

[hbm70598-bib-0025] Driesen, N. R. , G. McCarthy , Z. Bhagwagar , et al. 2013. “The Impact of NMDA Receptor Blockade on Human Working Memory‐Related Prefrontal Function and Connectivity.” Neuropsychopharmacology 38: 2613–2622. 23856634 10.1038/npp.2013.170 PMC3828532

[hbm70598-bib-0026] Dukart, J. , Š. Holiga , C. Chatham , et al. 2018. “Cerebral Blood Flow Predicts Differential Neurotransmitter Activity.” Scientific Reports 8: 4074. 29511260 10.1038/s41598-018-22444-0 PMC5840131

[hbm70598-bib-0027] Eickhoff, S. B. , K. E. Stephan , H. Mohlberg , et al. 2005. “A New SPM Toolbox for Combining Probabilistic Cytoarchitectonic Maps and Functional Imaging Data.” NeuroImage 25: 1325–1335. 15850749 10.1016/j.neuroimage.2004.12.034

[hbm70598-bib-0028] Ettinger, U. , D. H. Ffytche , V. Kumari , et al. 2008. “Decomposing the Neural Correlates of Antisaccade Eye Movements Using Event‐Related FMRI.” Cerebral Cortex 18: 1148–1159. 17728263 10.1093/cercor/bhm147

[hbm70598-bib-0029] Ettinger, U. , E. Faiola , A. M. Kasparbauer , et al. 2017. “Effects of Nicotine on Response Inhibition and Interference Control.” Psychopharmacology 234: 1093–1111. 28150023 10.1007/s00213-017-4542-8

[hbm70598-bib-0030] Ettinger, U. , I. Meyhöfer , M. A. Mehta , et al. 2018. “Effects of Lorazepam on Saccadic Eye Movements: The Role of Sex, Task Characteristics and Baseline Traits.” Journal of Psychopharmacology 32: 678–690. 29783905 10.1177/0269881118772450

[hbm70598-bib-0031] Faßbender, K. , K. Bey , J. V. Lippold , B. Aslan , R. Hurlemann , and U. Ettinger . 2021. “GABAergic Modulation of Performance in Response Inhibition and Interference Control Tasks.” Journal of Psychopharmacology 35: 1496–1509. 34278874 10.1177/02698811211032440

[hbm70598-bib-0032] Faßbender, K. , P. M. Baumert , M. W. M. Wintergerst , et al. 2023. “GABAergic Involvement in Selective Attention.” Journal of Cognitive Neuroscience 35: 976–989. 36976900 10.1162/jocn_a_01989

[hbm70598-bib-0033] Faul, F. , E. Erdfelder , A. Buchner , and A. G. Lang . 2009. “Statistical Power Analyses Using G*Power 3.1: Tests for Correlation and Regression Analyses.” Behavior Research Methods 41: 1149–1160. 19897823 10.3758/BRM.41.4.1149

[hbm70598-bib-0034] Fillmore, M. T. , C. R. Rush , T. H. Kelly , and L. Hays . 2001. “Triazolam Impairs Inhibitory Control of Behavior in Humans.” Experimental and Clinical Psychopharmacology 94: 363–404. 10.1037//1064-1297.9.4.363 11764012

[hbm70598-bib-0035] Ford, K. A. , H. C. Goltz , M. R. Brown , and S. Everling . 2005. “Neural Processes Associated With Antisaccade Task Performance Investigated With Event‐Related fMRI.” Journal of Neurophysiology 94: 429–440. 15728770 10.1152/jn.00471.2004

[hbm70598-bib-0036] Forsyth, A. E. M. , R. McMillan , J. Dukart , J. F. Hipp , and S. D. Muthukumaraswamy . 2021. “Effects of Ketamine and Midazolam on Simultaneous EEG/fMRI Data During Working Memory Processes.” Brain Topography 34: 863–880. 34642836 10.1007/s10548-021-00876-8

[hbm70598-bib-0037] Friedman, N. P. , and A. Miyake . 2004. “The Relations Among Inhibition and Interference Control Functions: A Latent‐Variable Analysis.” Journal of Experimental Psychology. General 133: 101–135. 14979754 10.1037/0096-3445.133.1.101

[hbm70598-bib-0038] Fuchs, A. F. , C. R. S. Kaneko , and C. A. Scudder . 1985. “Brainstem Control of Saccadic Eye Movements.” Annual Review of Neuroscience 8: 307–337. 10.1146/annurev.ne.08.030185.001515 3920944

[hbm70598-bib-0039] Green, J. F. , and D. J. King . 1998. “The Effects of Chlorpromazine and Lorazepam on Abnormal Antisaccade and No‐Saccade Distractibility.” Biological Psychiatry 44: 709–715. 9798074 10.1016/s0006-3223(97)00452-6

[hbm70598-bib-0040] Green, J. F. , D. J. King , and K. M. Trimble . 2000. “Antisaccade and Smooth Pursuit Eye Movements in Healthy Subjects Receiving Sertraline and Lorazepam.” Journal of Psychopharmacology 14: 30–36. 10757250 10.1177/026988110001400103

[hbm70598-bib-0041] Griffis, J. C. , A. S. Eikhetali , W. K. Burge , R. H. Chen , and K. M. Visscher . 2015. “Retinotopic Patterns of Background Connectivity Between V1 and Fronto‐Parietal Cortex Are Modulated by Task Demands.” Frontiers in Human Neuroscience 9: 338. 26106320 10.3389/fnhum.2015.00338 PMC4458688

[hbm70598-bib-0042] Hendriks, A. D. , F. D'Agata , L. Raimondo , et al. 2020. “Pushing Functional MRI Spatial and Temporal Resolution Further: High‐Density Receive Arrays Combined With Shot‐Selective 2D CAIPIRINHA for 3D Echo‐Planar Imaging at 7 T.” NMR in Biomedicine 33: e4281. 32128898 10.1002/nbm.4281 PMC7187278

[hbm70598-bib-0043] Hershey, T. , K. J. Black , J. Hartlein , et al. 2004. “Dopaminergic Modulation of Response Inhibition: An fMRI Study.” Cognitive Brain Research 20: 438–448. 15268921 10.1016/j.cogbrainres.2004.03.018

[hbm70598-bib-0044] Herweg, N. A. , B. Weber , A. Kasparbauer , et al. 2014. “Functional Magnetic Resonance Imaging of Sensorimotor Transformations in Saccades and Antisaccades.” NeuroImage 102: 848–860. 25173413 10.1016/j.neuroimage.2014.08.033

[hbm70598-bib-0045] Honey, R. A. E. , G. D. Honey , C. O'Loughlin , et al. 2004. “Acute Ketamine Administration Alters the Brain Responses to Executive Demands in a Verbal Working Memory Task: An fMRI Study.” Neuropsychopharmacology 29: 1203–1214. 15100698 10.1038/sj.npp.1300438 PMC3838946

[hbm70598-bib-0046] Hutton, S. B. 2008. “Cognitive Control of Saccadic Eye Movements.” Brain and Cognition 68: 327–340. 19028265 10.1016/j.bandc.2008.08.021

[hbm70598-bib-0047] Jamadar, S. D. , J. Fielding , and G. F. Egan . 2013. “Quantitative Meta‐Analysis of fMRI and PET Studies Reveals Consistent Activation in Fronto‐Striatal‐Parietal Regions and Cerebellum During Antisaccades and Prosaccades.” Frontiers in Psychology 4: 749. 24137150 10.3389/fpsyg.2013.00749 PMC3797465

[hbm70598-bib-0048] Kasparbauer, A. M. , N. Petrovsky , P. M. Schmidt , et al. 2019. “Effects of Nicotine and Atomoxetine on Brain Function During Response Inhibition.” European Neuropsychopharmacology 29: 235–246. 30552041 10.1016/j.euroneuro.2018.12.004

[hbm70598-bib-0049] Kimmig, H. , M. W. Greenlee , M. Gondan , M. Schira , J. Kassubek , and T. Mergner . 2001. “Relationship Between Saccadic Eye Movements and Cortical Activity as Measured by fMRI: Quantitative and Qualitative Aspects.” Experimental Brain Research 141: 184–194. 11713630 10.1007/s002210100844

[hbm70598-bib-0050] Kirby, K. N. 2009. “One‐Year Temporal Stability of Delay‐Discount Rates.” Psychonomic Bulletin & Review 16: 457–462. 19451368 10.3758/PBR.16.3.457

[hbm70598-bib-0051] Kuznetsova, A. , P. B. Brockhoff , and R. H. B. Christensen . 2017. “lmerTest Package: Tests in Linear Mixed Effects Models.” Journal of Statistical Software 82: 1–26.

[hbm70598-bib-0052] Logue, S. F. , and T. J. Gould . 2014. “The Neural and Genetic Basis of Executive Function: Attention, Cognitive Flexibility, and Response Inhibition.” Pharmacology, Biochemistry, and Behavior 123: 45–54. 23978501 10.1016/j.pbb.2013.08.007 PMC3933483

[hbm70598-bib-0053] McCartan, D. , R. Bell , J. F. Green , et al. 2001. “The Differential Effects of Chlorpromazine and Haloperidol on Latent Inhibition in Healthy Volunteers.” Journal of Psychopharmacology 15: 96–104. 11448094 10.1177/026988110101500211

[hbm70598-bib-0054] McDowell, J. E. , K. A. Dyckman , B. P. Austin , and B. A. Clementz . 2008. “Neurophysiology and Neuroanatomy of Reflexive and Volitional Saccades: Evidence From Studies of Humans.” Brain and Cognition 68: 255–270. 18835656 10.1016/j.bandc.2008.08.016 PMC2614688

[hbm70598-bib-0055] Mehta, M. A. , A. M. Owen , B. J. Sahakian , N. Mavaddat , J. D. Pickard , and T. W. Robbins . 2000. “Methylphenidate Enhances Working Memory by Modulating Discrete Frontal and Parietal Lobe Regions in the Human Brain.” Journal of Neuroscience 20: RC65. 10704519 10.1523/JNEUROSCI.20-06-j0004.2000 PMC6772505

[hbm70598-bib-0056] Milner, A. D. , and M. A. Goodale . 1992. “Separate Visual Pathways for Perception and Action.” Trends in Neurosciences 15: 20–25. 1374953 10.1016/0166-2236(92)90344-8

[hbm70598-bib-0057] Munoz, D. P. , and S. Everling . 2004. “Look Away: The Anti‐Saccade Task and the Voluntary Control of Eye Movement.” Nature Reviews. Neuroscience 5: 218–228. 14976521 10.1038/nrn1345

[hbm70598-bib-0058] Nieto‐Castanon, A. 2020. Handbook of Functional Connectivity Magnetic Resonance Imaging Methods in CONN. Hilbert Press.

[hbm70598-bib-0059] Nørgaard, M. , V. Beliveau , M. Ganz , et al. 2021. “A High‐Resolution In Vivo Atlas of the Human Brain's Benzodiazepine Binding Site of GABA_A_ Receptors.” NeuroImage 232: 117878. 33610745 10.1016/j.neuroimage.2021.117878 PMC8256681

[hbm70598-bib-0060] Paulus, M. P. , J. S. Feinstein , G. Castillo , A. N. Simmons , and M. B. Stein . 2005. “Dose‐Dependent Decrease of Activation in Bilateral Amygdala and Insula by Lorazepam During Emotion Processing.” Archives of General Psychiatry 62: 282–288. 15753241 10.1001/archpsyc.62.3.282

[hbm70598-bib-0061] Pierce, J. E. , and J. E. Mcdowell . 2016. “Modulation of Cognitive Control Levels via Manipulation of Saccade Trial‐Type Probability Assessed With Event‐Related BOLD fMRI.” Journal of Neurophysiology 115: 763–772. 26609113 10.1152/jn.00776.2015

[hbm70598-bib-0062] Ploner, C. J. , B. M. Gaymard , S. Rivaud‐Péchoux , and C. Pierrot‐Deseilligny . 2005. “The Prefrontal Substrate of Reflexive Saccade Inhibition in Humans.” Biological Psychiatry 57: 1159–1165. 15866556 10.1016/j.biopsych.2005.02.017

[hbm70598-bib-0063] Polli, F. E. , J. J. Barton , M. S. Cain , K. N. Thakkar , S. L. Rauch , and D. S. Manoach . 2005. “Rostral and Dorsal Anterior Cingulate Cortex Make Dissociable Contributions During Antisaccade Error Commission.” Proceedings of the National Academy of Sciences of the United States of America 102: 15700–15705. 16227444 10.1073/pnas.0503657102 PMC1255733

[hbm70598-bib-0064] Reilly, J. L. , R. Lencer , J. R. Bishop , S. Keedy , and J. A. Sweeney . 2008. “Pharmacological Treatment Effects on Eye Movement Control.” Brain and Cognition 68: 415–435. 19028266 10.1016/j.bandc.2008.08.026 PMC3159189

[hbm70598-bib-0065] Robbins, T. W. , and A. F. T. Arnsten . 2009. “The Neuropsychopharmacology of Fronto‐Executive Function: Monoaminergic Modulation.” Annual Review of Neuroscience 32: 267–287. 10.1146/annurev.neuro.051508.135535 PMC2863127 19555290

[hbm70598-bib-0066] Saari, T. I. , M. Uusi‐Oukari , J. Ahonen , and K. T. Olkkola . 2011. “Enhancement of GABAergic Activity: Neuropharmacological Effects of Benzodiazepines and Therapeutic Use in Anesthesiology.” Pharmacological Reviews 63: 243–267. 21245208 10.1124/pr.110.002717

[hbm70598-bib-0067] Sarkar, S. , S. Choudhury , N. Islam , et al. 2020. “Effects of Diazepam on Reaction Times to Stop and Go.” Frontiers in Human Neuroscience 14: 567177. 33132880 10.3389/fnhum.2020.567177 PMC7573484

[hbm70598-bib-0068] Sarmiento, L. F. , J. A. Ríos‐Flórez , H. A. Paez‐Ardila , et al. 2023. “Pharmacological Modulation of Temporal Discounting: A Systematic Review.” Health 11: 1046. 10.3390/healthcare11071046 PMC10093895 37046974

[hbm70598-bib-0069] Schmidt, P. M. , P. M. Baumert , K. Faßbender , and U. Ettinger . 2026. “Effects of Benzodiazepines on Saccadic Eye Movements: A Systematic Review and Meta‐Analysis.” Journal of Psychopharmacology. 10.1177/02698811261436602. 42080339

[hbm70598-bib-0070] Schunck, T. , A. Mathis , G. Erb , I. J. Namer , A. Demazières , and R. Luthringer . 2010. “Effects of Lorazepam on Brain Activity Pattern During an Anxiety Symptom Provocation Challenge.” Journal of Psychopharmacology 24: 701–708. 19460871 10.1177/0269881109104864

[hbm70598-bib-0071] Sparks, D. L. 2002. “The Brainstem Control of Saccadic Eye Movements.” Nature Reviews. Neuroscience 3: 952–964. 12461552 10.1038/nrn986

[hbm70598-bib-0072] Steffens, M. , C. Neumann , A. M. Kasparbauer , et al. 2018. “Effects of Ketamine on Brain Function During Response Inhibition.” Psychopharmacology 235: 3559–3571. 30357437 10.1007/s00213-018-5081-7

[hbm70598-bib-0073] Stirnberg, R. , and T. Stöcker . 2021. “Segmented K‐Space Blipped‐Controlled Aliasing in Parallel Imaging for High Spatiotemporal Resolution EPI.” Magnetic Resonance in Medicine 85: 1540–1551. 32936488 10.1002/mrm.28486

[hbm70598-bib-0074] Vehtari, A. , A. Gelman , and J. Gabry . 2017. “Practical Bayesian Model Evaluation Using Leave‐One‐Out Cross‐Validation and WAIC.” Statistics and Computing 27: 1413–1432.

[hbm70598-bib-0075] Vossel, S. , J. J. Geng , and G. R. Fink . 2014. “Dorsal and Ventral Attention Systems: Distinct Neural Circuits but Collaborative Roles.” Neuroscientist 20: 150–159. 23835449 10.1177/1073858413494269 PMC4107817

[hbm70598-bib-0076] Walter, S. A. , M. Forsgren , K. Lundengard , et al. 2016. “Positive Allosteric Modulator of GABA Lowers BOLD Responses in the Cingulate Cortex.” PLoS One 11: e0148737. 26930498 10.1371/journal.pone.0148737 PMC4773017

[hbm70598-bib-0077] Whitfield‐Gabrieli, S. , and A. Nieto‐Castanon . 2012. “Conn: A Functional Connectivity Toolbox for Correlated and Anticorrelated Brain Networks.” Brain Connectivity 2: 125–141. 22642651 10.1089/brain.2012.0073

[hbm70598-bib-0078] Zhang, R. , X. Geng , and T. M. C. Lee . 2017. “Large‐Scale Functional Neural Network Correlates of Response Inhibition: An fMRI Meta‐Analysis.” Brain Structure & Function 222: 3973–3990. 28551777 10.1007/s00429-017-1443-x PMC5686258

