# Supplementary material for: GABAergic Modulation of Brain Function During Prosaccade and Antisaccade Eye Movements: Evidence From Ultra‐High‐Field fMRI
Source: Hum Brain Mapp. 2026 Jul 5;47(10):e70598. doi: 10.1002/hbm.70598 (PMC13334141; doi:10.1002/hbm.70598)
Supplement: Supplementary file 1 — Table S1: Model evaluation metrics for models with and without interaction terms. Table S2: Linear mixed models for oculomotor data. Table S3: Blood oxygen level dependent (BOLD) activation for the main effect of condition (pro‐ and antisaccades). Table S4: Blood oxygen level dependent (BOLD) activation for the antisaccade > prosaccade contrast. Table S5: Blood oxygen level dependent (BOLD) activation for the prosaccade > antisaccade contrast. Table S6: Blood oxygen level dependent (BOLD) activation for placebo vs. lorazepam across saccade conditions (extended version of Table 2). Table S7: Results from regression analyses on first‐level difference images (placebo‐lorazepam) for combined pro‐ and antisaccades > baseline (extended version of Table 4). Figure S1: Brain activation for the main effect of saccade condition. Figure S2: Brain activation for the antisaccade > prosaccade contrast. Figure S3: Brain activation for the prosaccade > antisaccade contrast. Figure S4: GABAA receptor density within the BZD‐modulated network. [file HBM-47-e70598-s001.docx]

**GABAergic Modulation of Brain Function during Prosaccade and Antisaccade Eye Movements: Evidence from Ultra-High-Field fMRI**

**– Supplementary Material**

Paulina Quint (1), Pia-Magdalena Schmidt (1), Sarah Mackert (2), Behrem Aslan (2), Matthias Guth (2), Leon von der Emde (3), Raphael Lechtenboehmer (3), Kaja Faßbender (1), Philine M. Baumert (1), Birgit Stoffel-Wagner (4), Ramona Dolscheid-Pommerich (4), Rüdiger Stirnberg (5), Tony Stöcker (5), Ulrich Ettinger (1)*

(1) Department of Psychology, University of Bonn, Bonn, Germany

(2) Department of Psychiatry and Psychotherapy, University Hospital Bonn, Bonn, Germany

(3) Department of Ophthalmology, University Hospital Bonn, Bonn, Germany

(4) Institute of Clinical Chemistry and Clinical Pharmacology, University Hospital Bonn, Bonn, Germany

(5) German Center for Neurodegenerative Diseases (DZNE), Bonn, Germany

*Corresponding author:

Ulrich Ettinger
Department of Psychology
University of Bonn
Kaiser-Karl-Ring 9
53111 Bonn
Germany
Email: [ulrich.ettinger@uni-bonn.de](mailto:ulrich.ettinger@uni-bonn.de)
Phone: +49 228 734208

**Supplementary Methods**

**Selection Criteria for Participants**

Participants were required to be aged 18-40 years, to be free of psychiatric and neurological conditions, right-handed, non-smoking, to have normal or corrected-to-normal vision and sufficient German language skills. Exclusion criteria were consumption of any medication (with the exception of oral contraceptives in women and thyroid medications), current diagnosis of physical condition, in particular respiratory dysfunction, hereditary galactose intolerance, complete lack of lactase, or glucose-galactose-malabsorption, current diagnosis of psychiatric or neurological condition, past diagnosis of a psychiatric or neurological condition, blood pressure below 100/60 or over 140/90, resting pulse < 60 per minute or > 100 per minute, body mass index (BMI) < 18 or > 29 for men, BMI < 19 or > 30 for women, injuries to the eyes or eye disease, consumption of nicotine or drugs, positive urine drug test, positive alcohol breathalyser test, earlier consumption of lorazepam or other benzodiazepines (lifetime), known allergic reactions to medications, claustrophobia or having had a panic attack in a small space, impairments that prevent lying still on the back for 60-90 minutes, positive urine pregnancy test, breastfeeding or not using effective contraceptives for the duration of at least one cycle for women, and extensive tattoos or metal parts in the body (e.g. implants, prostheses, medication pumps, pace makers, earrings, intrauterine devices or piercings).

**Study Procedure**

Participants were recruited via flyers on the University of Bonn campus and online advertisements. They were invited to complete a short online questionnaire (approx. 5 min), in which basic inclusion and exclusion criteria were listed. If eligible, they were invited to an in-person screening at the University of Bonn (approx. 1 hour), where they provided written, informed consent and inclusion and exclusion criteria were checked in detail. Subsequently, a screening for psychiatric disorders was conducted using the German version 5.0.0 of the Mini International Neuropsychiatric Interview (MINI) (Sheehan et al., 1998) German translation by (Ackenheil et al., 1999) as well as a urine drug test screening for amphetamine, benzodiazepines, morphine, opiates and cannabinoids (Drug-Screen Multi 4P Test, nal von minden GmbH, Moers, Germany) and, for women only, a urine pregnancy test (NADAL® hCG Test, nal von minden GmbH, Moers, Germany). At the end of the screening, participants practiced short versions of the tasks they were to perform in the scanner. The shortened version of the saccade task consisted of 6 blocks (approx. 3 min), with no data collected. Participants meeting all inclusion and no exclusion criteria were then invited to take part in two MRI sessions at the German Center for Neurodegenerative Diseases (DZNE), Bonn.

The first MRI session was conducted approximately two months after the in-person screening (*M* = 57.2 days, *SD* = 32.2, range from 10-154 days). The second MRI session was conducted 1 week later, with a few deviations due to technical problems or health-related interruptions (*M* = 13.6 days, *SD* = 20.9, *Md* = 7 days, range from 6-98 days). The procedure of both MRI sessions was almost identical, except for the drug condition. Participants arrived in the morning between 8 and 11 AM and first underwent an alcohol breath test (ACE AL5500 plus). Female participants further underwent another urine pregnancy test (no participant had to be excluded). In a brief medical consultation with a physician, participants were then informed about MRI-related safety aspects and provided written, informed consent. Afterwards, a capsule containing either 1mg lorazepam (Tavor®, Pfizer) or a placebo (P-Tabletten weiß 7 mm Lichtenstein, Zentiva) was administered orally to participants with the instruction to only consume water from this point onward until the end of the scanning procedure, followed by a waiting period of approximately 80 minutes, to allow task onset two hours after administration (Saari et al., 2011). Participants were then equipped with earplugs as well as a pulse oximeter and a breathing belt and positioned into the MRI scanner. The scanning procedure started with a structural T1 scan and was followed by three eye movement tasks, of which the current saccade task was performed first, approximately 120 minutes after capsule administration (*M* = 119 min, *SD* = 9 min). The remaining tasks will be reported elsewhere. Total scan time was about 1 hour.

Four MRI sessions had to be terminated prior to data collection due to technical issues and one session due to nausea experienced by the participant inside the scanner. After completing the tasks, participants were taken out of the scanner to an examination room, where they completed a short online questionnaire, including visual analogue rating scales (VAS) asking for their current mood state (Bond and Lader, 1974) as well as an item asking participants to guess the substance they believed they had received. Female participants were further asked to report the current phase of their menstrual cycle. Afterwards, blood pressure and pulse of participants was measured (interval between capsule administration and measurement: *M* = 175 min, *SD* = 12 min) and a blood sample was collected using a S-Monovette® tube (Sarstedt, Nümbrecht, Germany) with K3 EDTA as an anticoagulant (interval between capsule administration and blood sample: *M* = 186 min, *SD* = 14 min).

Participants were allowed to leave if they felt able to, and were instructed to not consume alcohol or operate heavy machinery for the rest of the day. They were compensated with either 80€ or course credits (for psychology students). Blood samples were subsequently centrifuged for 10 min at 1000g and approximately 500 µl plasma was pipetted and stored frozen before analysis at the central laboratory of the University Hospital Bonn. Quantitative analysis of lorazepam was performed using [liquid chromatography combined with mass spectrometry](https://www.sciencedirect.com/topics/medicine-and-dentistry/liquid-chromatography-mass-spectrometry) (LC-MS/MS) consisting of a LC-20 UFCL (Shimadzu, Kyoto, Japan) and a Triple Quad 4500 (AB Sciex, Framingham, MA, USA). Plasma concentrations were determined by a commercially available assay Chromsystems-MassTox® Benzodiazepines 2 (Chromsystems Instruments and Chemicals GmbH, Graefelfing, Germany).

**Saccade Task**

Initial distance from monitor to eye was approximately 2650mm (first six participants; three each in the PLC/LOR condition and three in the LOR/PLC condition) but was later changed to 2075mm (all others), in order to increase the visual angle of the target steps and thus improve eye-tracking data quality. Resulting variations were accounted for in the calculation of amplitude-based parameters.

The saccade task comprised five 30-second blocks for each condition of prosaccades, antisaccades, and fixation, presented in randomized order. At the beginning of the task, detailed instructions were presented to the participants. For prosaccades, they were instructed to follow the stimulus with their eyes as fast and accurately as possible, whereas for antisaccades they were instructed to look in the mirror opposite direction after the stimulus moved to the periphery, again as fast and accurately as possible. Each block was further preceded by a short instruction (2 seconds) about the condition to be performed in the upcoming block. Saccade blocks consisted of 10 trials each, with each trial starting with a central stimulus (0.42° [0.33°] diameter; values in square brackets refer to initial distance from monitor to eye) presented on a black (RGB: 0,0,0) background. Stimulus color was either blue (RGB: 0,0,255) for antisaccades and yellow (RGB: 255,255,0) for prosaccades or vice versa, with color-task mappings counterbalanced across participants but fixed within a given participant. After a random duration between 1100 and 1900 ms, the stimulus (RGB: 255, 255, 255) stepped horizontally to the left or right (randomized, ± 6.6° [5.17°]) and remained there until the full trial duration of 3000 ms was reached. Fixation blocks consisted of a continuously presented central white stimulus (0.42° [0.33°] diameter, RGB: 255,255,255).

**Eye Movement Data Acquisition**

Eye movements were recorded using an MR-compatible video-based combined pupil and corneal reflection eye tracker (EyeLink 1000, SR Research Ltd., Kanata, ON, Canada) with a sampling rate of 1000 Hz. By default, the right eye was tracked in 56 out of 62 sessions (90.3%), except for six sessions where the left eye was recorded as an attempt to improve data quality (9.7%). A five-point horizontal-vertical calibration was performed before the task (calibration coordinates: [800,600], [800,102], [800,1098], [96,600], [1504,600]; 4.57° [3.58°] vertical distance and 7.73° [6.07°] horizontal distance to screen center). The calibration stimulus was a white circle (RGB: 255,255,255) filled with a smaller black (RGB: 0,0,0) circle on black (RGB: 0,0,0) background. The eye-tracker was fixated above the head coil and recorded pupil and corneal reflection signals through a hot mirror, reflecting infrared light and allowing visible light to pass, ensuring that participants were able to see the monitor. Light in the scanner room was switched off during the tasks.

**Oculomotor Data Analysis**

Preprocessing of oculomotor data was performed with EyeLink Data Viewer software package (SR Research Ltd., version 4.4.1) and R (version 4.4.2), using RStudio (version 2025.05.0). Standard settings of EyeLink Data Viewer were used to detect saccades, except for an increased minimum amplitude threshold (2°) to account for relatively noisy data. For each trial, the first saccade after target onset was included in the analysis, if deviation of the saccade starting point from the horizontal target position did not exceed 250 pixels and there was no signal loss between target and saccade onset. Start and end points of saccades were adjusted manually when necessary. Trials with signal loss after saccade onset and before saccade offset were included in the analysis of latency and direction errors, while analysis of peak velocity and amplitude was based on the smaller proportion of trials with full saccade signal. Amplitude gain was calculated as the ratio of saccade amplitude to target amplitude. For each participant, trials with outlier values on peak velocity, amplitude gain and latency (based on 1.5 times the interquartile range) were excluded. Peak velocity was corrected for amplitude by calculating the ratio of peak velocity to amplitude given the well-known main sequence relationship (Martinez-Conde et al., 2009). Only datasets with a minimum of seven valid trials per parameter were included for further analyses.

Linear mixed models were estimated separately for peak velocity, amplitude gain, latency and error rate, with drug (placebo, lorazepam) and condition (prosaccade, antisaccade) as fixed factors and subject as random factor using the lme4 package (version 1.1.36) (Bates et al., 2015). Due to limitations in data quality, we deviated from the preregistration at this point and analyzed only those key variables using linear mixed models. Visual inspection of Q-Q plots for model residuals revealed acceptable deviations from normal distribution for all parameters except for error rate, for which a log (x + 1) transformation was applied, resulting in a distribution closer to normal. *P*-values were obtained via Satterthwaite's degrees of freedom method using the lmerTest package (version 3.1.3) (Kuznetsova et al., 2017). Comparisons of simpler with more complex models were performed via likelihood-ratio tests (χ²-tests based on model deviances), with models estimated using maximum likelihood (ML). Since none of the models benefited significantly from the inclusion of an interaction term between drug and condition, only results of the simpler models are reported. The final models were estimated using restricted maximum likelihood (REML). Parameters of model comparisons can be found in Table S2.

**MRI Data Analysis**

For second-level blood oxygen level dependent (BOLD) data analyses, we deviated from the preregistration and used a full factorial model instead of several *t*-tests, in order to simultaneously account for main and interaction effects within one model.

For the calculation of voxel overlap between the main effect of condition and the drug effects, masks corrected using two different approaches were deliberately compared (voxel-level family-wise error rate (FWE) corrected *p* < .05 with a minimum cluster size of 20 voxels for the main effect of condition; voxel-level threshold of *p* < .001 and a cluster-size threshold of FWE-corrected *p* < .05 for drug effects). This constitutes a conservative strategy for assessing voxel overlap, as a stricter correction was applied to the main effect of condition to provide a clearer and more interpretable representation of activated regions. Applying the more liberal correction to the main effect of condition would have led to a slightly larger extent of overlap.

All MRI figures were created using MRIcroGL 1.2.20220720.

**Functional Connectivity Analysis**

Connectivity analysis was performed using CONN 22.v2407 (Whitfield-Gabrieli and Nieto-Castanon, 2012). Anatomical data were normalized into standard MNI space, segmented into grey matter, white matter and cerebral spinal fluid (CSF), and resampled to 1 mm isotropic voxels using SPM unified segmentation and normalization algorithm (Ashburner, 2007; Ashburner and Friston, 2005) with the default IXI-549 tissue probability map template.

In addition, functional data were denoised using a standard denoising pipeline (Nieto-Castanon, 2020) including the regression of potential confounding effects characterized by white matter timeseries (5 CompCor noise components), CSF timeseries (5 CompCor noise components), session and task effects and their first order derivatives (20 factors), and linear trends (2 factors) within each functional run, followed by bandpass frequency filtering of the BOLD timeseries (Hallquist et al., 2013) between 0.01 Hz and 0.1 Hz. CompCor (Behzadi et al., 2007; Chai et al., 2012) noise components within white matter and CSF were estimated by computing the average BOLD signal as well as the largest principal components orthogonal to the BOLD average within each subject's eroded segmentation masks. From the number of noise terms included in this denoising strategy, the effective degrees of freedom of the BOLD signal after denoising were estimated to be 138.2 across all subjects (Nieto-Castanon, 2025).

For first-level analyses, psychophysiological interaction (PPI) analyses were used to study changes in functional connectivity across conditions. Seed-to-voxel generalized PPI (gPPI) analyses were performed with seed regions in 8 regions of interest (ROIs; bilateral V1, posterior IPS, anterior IPS, medial FEF). These regions are part of the dorsal visual pathway and are essential areas of the saccadic network (Ettinger et al., 2008; Jamadar et al., 2013; Milner and Goodale, 1992; Vossel et al., 2014). Coordinates for ROIs were extracted from Aichert and colleagues (Aichert et al., 2012), who used a very similar saccade task in an independent sample. For creation of each ROI, mean x-, y- and z-coordinates were calculated across the contrasts prosaccade vs. fixation and antisaccade vs. fixation reported by Aichert and colleagues (Aichert et al., 2012). A 10-mm sphere was then centered around the resulting coordinates. Separately for each pair of seed and target areas, a generalized psychophysiological interaction model (gPPI) (Friston et al., 1997; McLaren et al., 2012) was defined with seed BOLD signals as physiological factors, boxcar signals characterizing each individual task condition convolved with an SPM canonical hemodynamic response function as psychological factors, and the product of the two as psychophysiological interaction terms. Functional connectivity changes across conditions were characterized by the Fisher-transformed semipartial correlation coefficient of the PPI terms in each model.

Second-level analyses were then performed by estimating separate General Linear Models for each individual voxel. Paired *t*-tests were conducted to compare connectivity during combined pro- and antisaccade conditions between placebo and lorazepam. Voxel-level hypotheses were evaluated using multivariate parametric statistics with random-effects across subjects and sample covariance estimation across multiple measurements. Inferences were performed at the level of individual clusters. Cluster-level inferences were based on parametric statistics from Gaussian Random Field theory (Nieto-Castanon, 2020; Worsley et al., 1996). Results were thresholded using a voxel-level threshold of *p* < .001 and a cluster-size threshold of family-wise error (FWE) corrected *p* < .05.

**Associations with GABA_A_ Receptor Density**

Analyses of associations between BOLD data and GABA_A_ receptor density from a publicly available atlas (Nørgaard et al., 2021) were performed using Matlab 2024b. We first compared GABA_A_ receptor densities (pmol/ml) between brain regions with and without significant drug effects. Results from the whole-brain analysis were used to extract a binary mask from the placebo > lorazepam contrast (Table 2, Figure 3A), comprising all areas with a significant effect. By applying this mask to the GABA_A_ receptor density atlas by Nørgaard and colleagues (Nørgaard et al., 2021), mean density values within and outside masked regions were obtained and a two-sample *t*-test with Welch correction, accounting for unequal variances, was calculated.

Second, given clear evidence of higher receptor density values in cortical areas, compared to subcortical areas and brainstem (Nørgaard et al., 2021), we further compared receptor densities between regions with and without significant drug effects, but this time restricted to cortical areas. A cortex mask was built using the WFU pick atlas software (Lancaster et al., 2000, 1997; Maldjian et al., 2004, 2003) and regions with significant drug effects were excluded from the mask. Mean density values within this cortex mask were then calculated and compared to mean values within the previously used mask based on drug effect using a two-sample *t*-test with Welch correction.

Third, Pearson correlations between GABA_A_ receptor density and the strength of lorazepam effect on BOLD were computed at voxel-level. Therefore, *t*-values from the placebo > lorazepam contrast as well as GABA_A_ receptor density values were extracted for each voxel within the binary effect mask as well as for the whole brain.

**Analysis of Signal Loss as Proxy for Eyelid Closure**

We additionally assessed whether reductions in BOLD signal under lorazepam are partially attributable to more frequent eyelid closure. Although a significant amount of oculomotor signal loss stems from technical issues, it can be used as proxy for eyelid closure. For each participant and session with oculomotor data available, the total duration of signal loss during pro- and antisaccade trials was determined. A paired *t*-test was performed to assess whether the observed increase in eyelid closure during lorazepam was reflected in the data. Change scores (placebo - lorazepam) for the duration of signal loss across saccade conditions were then calculated (*N* = 28). These change scores were entered into second-level multiple regression analyses together with difference BOLD images (placebo – lorazepam), which were derived from first-level contrasts (combined pro- and antisaccades > baseline). Results were thresholded using a voxel-level threshold of *p* < .001 and a cluster-size threshold of family-wise error (FWE) corrected *p* < .05.

**Comparison of GABA_A_ Receptor Density Across Paradigms**

In order to assess whether increased GABA_A_ receptor density in regions associated with the employed saccade task is specific to this paradigm, we performed analogous analyses for the intertemporal choice paradigm, which serves as a representative paradigm with no evidence for behavioral BZD effects (Sarmiento et al., 2023), using Matlab 2024b. Therefore, masks for the ventral striatum and ventromedial prefrontal cortex were combined. These regions were identified as representative neural markers for paradigms based on subjective valuation (Bartra et al., 2013). The combined mask and the binary mask from the placebo > lorazepam contrast, extracted from our whole-brain analysis (Table 2, Figure 3A), were then applied to the GABA_A_ receptor density atlas by Nørgaard and colleagues (Nørgaard et al., 2021). Mean density values within both masked regions were then compared using a two-sample *t*-test, assuming equal variances (two-sample *F*-test for equal variances, *p* > .05). In a second step, mean density values within the mask of regions associated with subjective value were compared to the remaining brain areas using a two-sample *t*-test with Welch correction, accounting for unequal variances (two-sample *F*-test for equal variances, *p* < .05).

**Supplementary Results**

**Participants**

Participants had an average height of 171.72 cm (*SD* = 8.28, range 158.0-187.0), an average weight of 68.19 kg (*SD* = 9.36, range 53.6-92.8), and an average BMI of 23.14 (*SD* = 2.89, range 18.7-30.0). In 56 of 75 sessions (74.67 %), participants were able to correctly identify the current drug condition. No significant differences between placebo and lorazepam sessions were found for systolic (*t*(35) = -0.23, *p* = .816) or diastolic (*t*(35) = -1.20, *p* = .240) blood pressure. Under lorazepam, participants showed a significantly higher pulse (*M* = 70.28, *SD*= 10.47) than under placebo (*M*= 64.78, *SD*= 8.73; *t*(35) = -3.57, *p*< .01, *d* = -0.60). Lorazepam plasma levels were significantly higher for lorazepam sessions (*M* = 11.84 μg/l, *SD* = 1.58) than for placebo (*M* = 10 μg/l, *SD* = 0; *t*(34) = -6.89, *p* < .001, *d* = -1.16). Note that plasma levels in the placebo condition were all below the quantification limit of 10 μg/l and were conservatively set to 10 μg/l for statistical analyses. For VAS, participants had significantly higher values for alertness (indicating less alertness) under lorazepam (*M* = 58.02, SD = 13.61) than under placebo (*M* = 38.62, *SD* = 17.94; *t*(35) = -6.67, *p* < .001, *d* = -1.11). Similarly, values on contentedness were significantly higher (indicating less contentedness) under lorazepam (*M* = 36.51, *SD* = 11.76) than under placebo (*M* = 28.19, *SD* = 13.35; *t*(35) = -3.78, *p* < .001, *d* = -0.63). No significant differences were found for calmness (*t*(35) = 0.39, *p* = .696).

**Regression Analyses for Plasma Levels and VAS Subscales**

For lorazepam plasma levels, change scores (placebo – lorazepam) were significantly negatively associated with activation during placebo compared to lorazepam condition in several regions, including left precentral and postcentral gyrus, right insula and intracalcarine cortex as well as bilateral precuneus, posterior cingulate gyrus, lingual gyrus and central opercular cortex (Table S7). Greater increases in lorazepam plasma levels from placebo were therefore associated with greater activation differences between placebo and lorazepam in these regions.

For VAS contentedness change scores (placebo – lorazepam), significant positive associations with neural changes were found for right central opercular and insular cortex (Table S7). Greater reductions in contentedness (i.e., higher scale values under lorazepam) were therefore associated with less activation differences between placebo and lorazepam in these regions.

**Analysis of Signal Loss as Proxy for Eyelid Closure**

EyeLink signal loss under lorazepam (*M* = 70 089.04 ms, *SD* = 57 138.46 ms) was significantly higher than under placebo (*M* = 33 977.96 ms, *SD* = 46 584.31 ms; *t*(27) = -3.08, *p* < .01, *d* = -0.69). However, regression analyses did not reveal any significant clusters where lorazepam effects could be predicted by the amount of signal loss (voxel-level threshold set to *p* < .001, uncorrected; cluster-level threshold set to *p* < .05, FWE-corrected). Thus, increased eye-tracking signal loss during lorazepam compared to placebo was unlikely to have caused reductions in BOLD signal for lorazepam compared to placebo.

**Comparison of GABA_A_ Receptor Density Across Paradigms**

GABA_A_ receptor density was significantly higher in regions with significant drug effects in this study (*M* = 636.56, *SD* = 356.75), compared to regions associated with subjective value in the meta-analysis (Bartra et al., 2013) (*M* = 393.28, *SD* = 359.29; *t*(95 450) = 57.15, *p* < .001, *d* = 0.68). However, GABA_A_ receptor density within regions associated with subjective value was significantly higher than density within remaining brain areas (*M* = 110.77, *SD* = 249.48; *t*(7 651.81) = 68.73, *p* < .001, *d* = 1.13).

| **Table S1** | | | | | | | | |
| --- | --- | --- | --- | --- | --- | --- | --- | --- |
| *Model evaluation metrics for models with and without interaction terms* | | | | | | | | |
| Model specification | Compared to model | Model Fit | | | | Model comparison | | |
|  |  | AIC | BIC | LL | df | Δdf | χ² | *p*-value |
| Peak Velocity: Drug + Condition |  | 1 045.04 | 1 058.31 | -517.52 | 5 |  |  |  |
| Peak Velocity: Drug * Condition | Peak Velocity: Drug + Condition | 1 046.78 | 1 062.70 | -517.39 | 6 | 1 | 0.264 | .607 |
| Peak Velocity, corrected: Drug + Condition |  | 612.12 | 625.39 | -301.06 | 5 |  |  |  |
| Peak Velocity, corrected: Drug * Condition | Peak Velocity, corrected: Drug + Condition | 613.87 | 629.79 | -300.93 | 6 | 1 | 0.249 | .618 |
| Amplitude Gain: Drug + Condition |  | -185.81 | -172.54 | 97.90 | 5 |  |  |  |
| Amplitude Gain: Drug * Condition | Amplitude Gain: Drug + Condition | -183.97 | -168.05 | 97.99 | 6 | 1 | 0.166 | .683 |
| Latency: Drug + Condition |  | 1 012.80 | 1 026.35 | -501.40 | 5 |  |  |  |
| Latency: Drug * Condition | Latency: Drug + Condition | 1 014.54 | 1 030.80 | -501.27 | 6 | 1 | 0.257 | .612 |
| Error Rate, log: Drug + Condition |  | 321.13 | 334.98 | -155.57 | 5 |  |  |  |
| Error Rate, log: Drug * Condition | Error Rate, log: Drug + Condition | 321.52 | 338.14 | -154.76 | 6 | 1 | 1.613 | .204 |
| *Note.* All models include random intercepts for subjects. | | | | | | | | |

| **Table S2**  *Linear mixed models for oculomotor data* | | | | | | |  |
| --- | --- | --- | --- | --- | --- | --- | --- |
| Linear mixed model for peak velocity | | | | | | |  |
|  | Fixed effects | | | | | |  |
|  | Estimate | Standard error | 95% CI | | *t* (df) | *p* |  |
| Intercept | 285.28 | 7.01 | 271.54, 299.02 | | 40.68 (46.13) | <.001 |  |
| Drug (lorazepam) | -24.33 | 5.19 | -34.51, -14.15 | | -4.69 (71.68) | <.001 |  |
| Condition (antisaccade) | -20.09 | 5.20 | -30.28, -9.91 | | -3.87 (71.48) | <.001 |  |
|  | Random Effect | | | | | |  |
|  | Variance | | | SD | | |  |
| Participant (Intercept) | 1 145.87 | | | 33.85 | | |  |
|  | Model fit | | | | | |  |
| R^2^ | Marginal | | | Conditional | | |  |
|  | 0.12 | | | 0.68 | | |  |
| Linear mixed model for corrected peak velocity | | | | | | |  |
|  | Fixed effects | | | | | |  |
|  | Estimate | Standard error | 95% CI | | *t*(df) | *p* |  |
| Intercept | 46.98 | 1.14 | 44.75, 49.21 | | 41.30 (38.26) | <.001 |  |
| Drug (lorazepam) | -2.63 | 0.58 | -3.76, -1.50 | | -4.55 (70.25) | <.001 |  |
| Condition (antisaccade) | -1.37 | 0.58 | -2.51, -0.24 | | -2.37 (70.16) | <.001 |  |
|  | Random Effect | | | | | |  |
|  | Variance | | | SD | | |  |
| Participant (Intercept) | 37.54 | | | 6.13 | | |  |
|  | Model fit | | | | | |  |
| R^2^ | Marginal | | | Conditional | | |  |
|  | 0.04 | | | 0.84 | | |  |
| Linear mixed model for amplitude gain | | | | | | |  |
|  | Fixed effects | | | | | |  |
|  | Estimate | Standard error | 95% CI | | *t*(df) | *p* |  |
| Intercept | 0.94 | 0.02 | 0.91, 0.97 | | 60.54 (84.83) | <.001 |  |
| Drug (lorazepam) | -0.02 | 0.02 | -0.06, 0.01 | | -1.33 (79.57) | 0.1859 |  |
| Condition (antisaccade) | 0.01 | 0.02 | -0.03, 0.04 | | 0.38 (79.18) | 0.7044 |  |
|  | Random Effect | | | | | |  |
|  | Variance | | | SD | | |  |
| Participant (Intercept) | 0.002 | | | 0.042 | | |  |
|  | Model fit | | | | | |  |
| R^2^ | Marginal | | | Conditional | | |  |
|  | 0.02 | | | 0.20 | | |  |
| Linear mixed model for latency | | | | | | | |
|  | | Fixed effects | | | | | |
|  | | Estimate | Standard error | 95% CI | | *t*(df) | *p* |
| Intercept | | 223.35 | 4.00 | 215.5, 231.2 | | 55.78 (62.29) | <.001 |
| Drug (lorazepam) | | 10.18 | 3.69 | 2.95, 17.4 | | 2.76 (81.43) | 0.0071 |
| Condition (antisaccade) | | 68.91 | 3.62 | 61.82, 76 | | 19.05 (78.55) | <.001 |
|  | | Random Effect | | | | | |
|  | | Variance | | | SD | | |
| Participant (Intercept) | | 266.26 | | | 16.32 | | |
|  | | Model fit | | | | | |
| R^2^ | | Marginal | | | Conditional | | |
|  | | 0.66 | | | 0.81 | | |
| Linear mixed model for error rate, log (x + 1) transformation | | | | | | | |
|  | | Fixed effects | | | | | |
|  | | Estimate | Standard error | 95% CI | | *t*(df) | *p* |
| Intercept | | 0.17 | 0.15 | -0.12, 0.46 | | 1.16 (91.51) | 0.2472 |
| Drug (lorazepam) | | 0.31 | 0.16 | 0, 0.62 | | 1.96 (93.33) | 0.0524 |
| Condition (antisaccade) | | 2.32 | 0.15 | 2.02, 2.62 | | 14.99 (86.29) | <.001 |
|  | | Random Effect | | | | | |
|  | | Variance | | | SD | | |
| Participant (Intercept) | | 0.18 | | | 0.42 | | |
|  | | Model fit | | | | | |
| R^2^ | | Marginal | | | Conditional | | |
|  | | 0.61 | | | 0.69 | | |

*Note*. *P*-values were calculated via Satterthwaite's degrees of freedom method. Confidence Intervals were calculated via the Wald method. Model equation: Peak velocity ~ 1 + Drug + Condition + (1|Subject).

| **Table S3**  *Blood oxygen level dependent (BOLD) activation for the main effect of condition (pro- and antisaccades)* | | | | | |  |
| --- | --- | --- | --- | --- | --- | --- |
| Anatomical label *(functional label)* | Cluster size | *F*-value | MNI coordinates | | | |
|  |  |  | x | y | z | |
| R Superior Frontal Gyrus *(medial FEF)* | 28 250 | 190.51 | 27 | -5 | 52 | |
| R Superior Frontal Gyrus *(medial FEF)* |  | 166.18 | 26 | -1 | 59 | |
| R Paracingulate Gyrus |  | 86.55 | 6 | 14 | 42 | |
| R Paracingulate Gyrus *(preSMA)* |  | 86.32 | 8 | 11 | 47 | |
| L Paracingulate Gyrus |  | 82.57 | -2 | 9 | 47 | |
| R Precentral Gyrus |  | 82.39 | 54 | 10 | 25 | |
| R Frontal Operculum Cortex |  | 81.91 | 43 | 17 | 2 | |
| R Juxtapositional Lobule Cortex |  | 78.78 | 3 | 4 | 54 | |
| R Precentral Gyrus *(lateral FEF)* |  | 78.40 | 42 | 0 | 50 | |
| R Precentral Gyrus *(lateral FEF)* |  | 70.79 | 47 | 3 | 52 | |
| R Frontal Operculum Cortex |  | 59.85 | 34 | 22 | 8 | |
| R Inferior Frontal Gyrus, pars opercularis |  | 57.29 | 51 | 9 | 12 | |
| R Inferior Frontal Gyrus, pars opercularis |  | 54.50 | 55 | 13 | 12 | |
| R Precentral Gyrus *(lateral FEF)* |  | 37.16 | 54 | 8 | 40 | |
| R Lateral Occipital Cortex, superior division (SPL) | 72 376 | 177.18 | 17 | -68 | 60 | |
| R Precuneus (SPL) |  | 173.15 | 5 | -53 | 58 | |
| L Lateral Occipital Cortex, superior division (SPL) |  | 169.91 | -17 | -63 | 62 | |
| R Precuneus (SPL) |  | 169.05 | 9 | -61 | 62 | |
| R Lateral Occipital Cortex, superior division (SPL) |  | 169.02 | 20 | -63 | 58 | |
| R Precuneus (SPL) |  | 152.84 | 7 | -57 | 62 | |
| L Lateral Occipital Cortex, superior division (SPL) |  | 149.19 | -17 | -64 | 56 | |
| R Supramarginal Gyrus, posterior division (IPS) |  | 130.55 | 36 | -38 | 42 | |
| L Precuneus (SPL) |  | 121.97 | -8 | -53 | 55 | |
| R Lateral Occipital Cortex, superior division (SPL) |  | 114.31 | 20 | -69 | 49 | |
| R Superior Parietal Lobule |  | 102.27 | 19 | -55 | 63 | |
| R Superior Parietal Lobule |  | 95.18 | 24 | -56 | 65 | |
| L Superior Parietal Lobule (IPS) |  | 88.31 | -33 | -43 | 41 | |
| R Supramarginal Gyrus, anterior division (IPL) |  | 86.69 | 60 | -22 | 44 | |
| R Supramarginal Gyrus, posterior division (IPL) |  | 76.46 | 62 | -35 | 39 | |
| R Supramarginal Gyrus, posterior division (IPL) |  | 74.64 | 63 | -41 | 36 | |
| R Occipital Pole *(V3v)* | 3 556 | 171.71 | 32 | -93 | -7 | |
| L Middle Frontal Gyrus *(medial FEF)* | 8 706 | 160.35 | -27 | -5 | 50 | |
| L Superior Frontal Gyrus *(medial FEF)* |  | 150.32 | -24 | -4 | 58 | |
| L Precentral Gyrus |  | 54.22 | -49 | 4 | 39 | |
| L Superior Frontal Gyrus |  | 51.43 | -18 | -4 | 74 | |
| L Precentral Gyrus |  | 30.42 | -41 | -1 | 37 | |
| L Occipital Pole *(V3v)* | 2 418 | 158.88 | -29 | -95 | -13 | |
| L Occipital Pole *(V3v)* |  | 152.93 | -25 | -97 | -14 | |
| L Frontal Operculum Cortex | 3 685 | 71.52 | -39 | 15 | 2 | |
| L Inferior Frontal Gyrus, pars opercularis |  | 41.71 | -52 | 11 | 3 | |
| L Inferior Frontal Gyrus, pars opercularis |  | 33.34 | -52 | 10 | 18 | |
| L Precuneus | 14 712 | 66.82 | -8 | -54 | 11 | |
| Cingulate Gyrus, posterior division |  | 66.61 | 0 | -37 | 35 | |
| L Precuneus |  | 64.33 | -5 | -63 | 21 | |
| R Precuneus |  | 63.44 | 8 | -52 | 12 | |
| Cingulate Gyrus, posterior division |  | 57.53 | 0 | -43 | 32 | |
| L Cingulate Gyrus, posterior division |  | 53.90 | -4 | -53 | 23 | |
| R Precuneus |  | 50.88 | 4 | -54 | 27 | |
| R Frontal Pole | 3 814 | 61.47 | 31 | 40 | 26 | |
| R Frontal Pole |  | 41.56 | 38 | 40 | 32 | |
| R Middle Frontal Gyrus |  | 36.85 | 35 | 34 | 37 | |
| R Frontal Pole | 505 | 51.03 | 2 | 63 | -4 | |
| R Lateral Occipital Cortex, superior division (IPL) | 952 | 48.65 | 48 | -65 | 30 | |
| L Lateral Occipital Cortex, superior division (IPL) | 2 550 | 48.16 | -47 | -67 | 34 | |
| L Lateral Occipital Cortex, superior division (IPL) |  | 46.71 | -43 | -72 | 42 | |
| L Lateral Occipital Cortex, superior division (IPL) |  | 43.55 | -39 | -68 | 36 | |
| L Lateral Occipital Cortex, superior division (IPL) |  | 40.18 | -48 | -67 | 40 | |
| L Lateral Occipital Cortex, superior division (IPS) |  | 33.63 | -38 | -70 | 47 | |
| L Lateral Occipital Cortex, superior division (IPL) |  | 33.40 | -46 | -74 | 34 | |
| L Lateral Occipital Cortex, superior division (IPL) |  | 32.39 | -45 | -66 | 46 | |
| R Cerebellum VI | 855 | 45.74 | 9 | -71 | -17 | |
| L Cerebellum VI |  | 38.95 | -6 | -73 | -21 | |
| R Cerebellum Vermis VI |  | 29.93 | 2 | -74 | -24 | |
| L Frontal Pole | 1 345 | 44.65 | -32 | 42 | 25 | |
| L Frontal Pole |  | 40.73 | -39 | 40 | 30 | |
| L Frontal Pole |  | 34.23 | -30 | 48 | 21 | |
| R Cerebellum VI | 140 | 36.25 | 28 | -59 | -33 | |
|  | 63 | 33.02 | -32 | -34 | -4 | |
| R Central Opercular Cortex | 228 | 32.98 | 36 | -18 | 21 | |
| R Central Opercular Cortex |  | 28.94 | 37 | -10 | 19 | |
| R Cerebellum | 52 | 31.27 | 2 | -76 | -33 | |
| R Middle Temporal Gyrus, temporooccipital part | 63 | 30.64 | 40 | -58 | 12 | |
| L Central Opercular Cortex | 41 | 30.20 | -43 | -11 | 19 | |
| L Central Opercular Cortex |  | 26.21 | -40 | -15 | 20 | |
| R Superior Frontal Gyrus | 44 | 28.95 | 21 | 27 | 49 | |
| R Occipital Pole *(V2)* | 23 | 28.73 | 13 | -98 | 10 | |
| L Superior Frontal Gyrus | 27 | 28.38 | -20 | 33 | 39 | |
| L Frontal Pole | 41 | 27.87 | -7 | 61 | 10 | |
| R Precentral Gyrus | 23 | 27.79 | 30 | -22 | 61 | |
| *Note.* Voxel-level threshold was set to *p* < .05, FWE-corrected. Cluster threshold was set to 20 voxels. FEF = frontal eye fields. PreSMA = presupplementary motor area. SPL = superior parietal lobule. IPS = intraparietal sulcus. IPL = inferior parietal lobule. V3v = ventral V3. | | | | | | |

| **Table S4**  *Blood oxygen level dependent (BOLD) activation for the antisaccade > prosaccade contrast* | | | | | |  |
| --- | --- | --- | --- | --- | --- | --- |
| Anatomical label *(functional label)* | Cluster size | *t*-value | MNI coordinates | | | |
|  |  |  | x | y | z | |
| R Superior Frontal Gyrus *(medial FEF)* | 30 130 | 13.80 | 27 | -5 | 52 | |
| R Superior Frontal Gyrus *(medial FEF)* |  | 12.89 | 26 | -1 | 59 | |
| R Paracingulate Gyrus |  | 9.30 | 6 | 14 | 42 | |
| R Paracingulate Gyrus *(preSMA)* |  | 9.29 | 8 | 11 | 47 | |
| L Paracingulate Gyrus |  | 9.091 | -2 | 9 | 47 | |
| R Precentral Gyrus |  | 9.08 | 54 | 10 | 25 | |
| R Frontal Operculum Cortex |  | 9.05 | 43 | 17 | 2 | |
| R Juxtapositional Lobule Cortex |  | 8.88 | 3 | 4 | 54 | |
| R Precentral Gyrus *(lateral FEF)* |  | 8.85 | 42 | 0 | 50 | |
| R Precentral Gyrus *(lateral FEF)* |  | 8.41 | 47 | 3 | 52 | |
| R Frontal Operculum Cortex |  | 7.74 | 34 | 22 | 8 | |
| R Inferior Frontal Gyrus, pars opercularis |  | 7.57 | 51 | 9 | 12 | |
| R Inferior Frontal Gyrus, pars opercularis |  | 7.38 | 55 | 13 | 12 | |
| R Precentral Gyrus |  | 6.10 | 54 | 8 | 40 | |
| R Lateral Occipital Cortex, superior division (SPL) | 77 206 | 13.31 | 17 | -68 | 60 | |
| R Precuneus (SPL) |  | 13.16 | 5 | -53 | 58 | |
| L Lateral Occipital Cortex, superior division (SPL) |  | 13.03 | -17 | -63 | 62 | |
| R Precuneus (SPL) |  | 13.00 | 9 | -61 | 62 | |
| R Lateral Occipital Cortex, superior division (SPL) |  | 13.00 | 20 | -63 | 58 | |
| R Precuneus (SPL) |  | 12.36 | 7 | -57 | 62 | |
| L Lateral Occipital Cortex, superior division (SPL) |  | 12.21 | -17 | -64 | 56 | |
| R Supramarginal Gyrus, posterior division (IPS) |  | 11.42 | 36 | -38 | 42 | |
| L Precuneus (SPL) |  | 11.04 | -8 | -53 | 55 | |
| R Lateral Occipital Cortex, superior division (IPL) |  | 10.69 | 20 | -69 | 49 | |
| R Superior Parietal Lobule |  | 10.11 | 19 | -55 | 63 | |
| R Superior Parietal Lobule |  | 9.76 | 24 | -56 | 65 | |
| L Superior Parietal Lobule (IPS) |  | 9.40 | -33 | -43 | 41 | |
| R Supramarginal Gyrus, anterior division (IPL) |  | 9.31 | 60 | -22 | 44 | |
| R Supramarginal Gyrus, posterior division (IPL) |  | 8.74 | 62 | -35 | 39 | |
| R Supramarginal Gyrus, posterior division (IPL) |  | 8.64 | 63 | -41 | 36 | |
| L Middle Frontal Gyrus *(medial FEF)* | 9 472 | 12.66 | -27 | -5 | 50 | |
| L Superior Frontal Gyrus *(medial FEF)* |  | 12.26 | -24 | -4 | 58 | |
| L Precentral Gyrus |  | 7.36 | -49 | 4 | 39 | |
| L Superior Frontal Gyrus |  | 7.17 | -18 | -4 | 74 | |
| L Precentral Gyrus |  | 5.51 | -41 | -1 | 37 | |
| L Frontal Operculum Cortex | 4 258 | 8.46 | -39 | 15 | 2 | |
| L Inferior Frontal Gyrus, pars opercularis |  | 6.46 | -52 | 11 | 3 | |
| L Inferior Frontal Gyrus, pars opercularis |  | 5.77 | -52 | 10 | 18 | |
| L Inferior Frontal Gyrus, pars opercularis |  | 5.16 | -49 | 8 | 27 | |
| R Frontal Pole | 4 253 | 7.84 | 31 | 40 | 26 | |
| R Frontal Pole |  | 6.45 | 38 | 40 | 32 | |
| R Middle Frontal Gyrus |  | 6.07 | 35 | 34 | 37 | |
| R Cerebellum VI | 1 205 | 6.76 | 9 | -71 | -17 | |
| L Cerebellum VI |  | 6.24 | -6 | -73 | -21 | |
| R Cerebellum Vermis Crus II |  | 5.59 | 2 | -76 | -33 | |
| R Cerebellum Vermis VI |  | 5.47 | 2 | -74 | -24 | |
| L Frontal Pole | 1 592 | 6.68 | -32 | 42 | 25 | |
| L Frontal Pole |  | 6.38 | -39 | 40 | 30 | |
| L Frontal Pole |  | 5.85 | -30 | 48 | 21 | |
| R Cerebellum VI | 193 | 6.02 | 28 | -59 | -33 | |
| R Cerebellum VI |  | 5.04 | 31 | -51 | -34 | |
| R Middle Temporal Gyrus, temporooccipital part | 110 | 5.54 | 40 | -58 | 12 | |
| R Occipital Pole *(V2)* | 54 | 5.36 | 13 | -98 | 10 | |
| NA (L Ventral Diencephalon) | 22 | 5.35 | -5 | -21 | -6 | |
| NA (R Ventral Diencephalon) | 28 | 5.23 | 6 | -21 | -7 | |
| *Note.* Voxel-level threshold was set to *p* < .05, FWE-corrected. Cluster threshold was set to 20 voxels. FEF = frontal eye fields. PreSMA = presupplementary motor area. SPL = superior parietal lobule. IPL = inferior parietal lobule. IPS = intraparietal sulcus. | | | | | | |

| **Table S5**  *Blood oxygen level dependent (BOLD) activation for the prosaccade > antisaccade contrast* | | | | | |  |
| --- | --- | --- | --- | --- | --- | --- |
| Anatomical label *(functional label)* | Cluster size | *t*-value | MNI coordinates | | | |
|  |  |  | x | y | z | |
| R Occipital Pole *(V3v)* | 3 683 | 13.10 | 32 | -93 | -7 | |
| L Occipital Pole *(V3v)* | 2 538 | 12.60 | -29 | -95 | -13 | |
| L Occipital Pole *(V3v)* |  | 12.37 | -25 | -97 | -14 | |
| L Precuneus | 15 924 | 8.17 | -8 | -54 | 11 | |
| Cingulate Gyrus, posterior division |  | 8.16 | 0 | -37 | 35 | |
| L Precuneus |  | 8.02 | -5 | -63 | 21 | |
| R Precuneus |  | 7.96 | 8 | -52 | 12 | |
| Cingulate Gyrus, posterior division |  | 7.58 | 0 | -43 | 32 | |
| L Cingulate Gyrus, posterior division |  | 7.34 | -4 | -53 | 23 | |
| R Precuneus |  | 7.13 | 4 | -54 | 27 | |
| R Frontal Pole | 579 | 7.14 | 2 | 63 | -4 | |
| R Lateral Occipital Cortex, superior division (IPL) | 1 192 | 6.98 | 48 | -65 | 30 | |
| L Lateral Occipital Cortex, superior division (IPL) | 2 914 | 6.94 | -47 | -67 | 34 | |
| L Lateral Occipital Cortex, superior division (IPL) |  | 6.83 | -43 | -72 | 42 | |
| L Lateral Occipital Cortex, superior division (IPL) |  | 6.60 | -39 | -68 | 36 | |
| L Lateral Occipital Cortex, superior division (IPL) |  | 6.34 | -48 | -67 | 40 | |
| L Lateral Occipital Cortex, superior division (IPS) |  | 5.80 | -38 | -70 | 47 | |
| L Lateral Occipital Cortex, superior division (IPL) |  | 5.78 | -46 | -74 | 34 | |
| L Lateral Occipital Cortex, superior division (IPL) |  | 5.69 | -45 | -66 | 46 | |
| L Hippocampus | 94 | 5.75 | -32 | -34 | -4 | |
| L Hippocampus |  | 5.06 | -35 | -30 | -7 | |
| R Central Opercular Cortex | 373 | 5.74 | 36 | -18 | 21 | |
| R Central Opercular Cortex |  | 5.38 | 37 | -10 | 19 | |
| L Central Opercular Cortex | 88 | 5.50 | -43 | -11 | 19 | |
| L Central Opercular Cortex |  | 5.12 | -40 | -15 | 20 | |
| R Superior Frontal Gyrus | 84 | 5.38 | 21 | 27 | 49 | |
| L Superior Frontal Gyrus | 67 | 5.33 | -20 | 33 | 39 | |
| L Frontal Pole | 133 | 5.28 | -7 | 61 | 10 | |
| R Precentral Gyrus | 55 | 5.27 | 30 | -22 | 61 | |
| L Superior Frontal Gyrus | 38 | 5.10 | -15 | 38 | 44 | |
| R Frontal Pole | 20 | 5.05 | 1 | 65 | 8 | |
| *Note.* Voxel-level threshold was set to *p* < .05, FWE-corrected. Cluster threshold was set to 20 voxels. V3v = ventral V3. IPL = inferior parietal lobule. IPS = intraparietal sulcus. | | | | | | |

| **Table S6**  *Blood oxygen level dependent (BOLD) activation for placebo vs. lorazepam across saccade conditions (extended version of Table 2)* | | | | | |  |
| --- | --- | --- | --- | --- | --- | --- |
| Anatomical label *(functional label)* | Cluster size | *t*-value | MNI coordinates | | | |
|  |  |  | x | y | z | |
| R Posterior Intraparietal Sulcus | 84 310 | 6.49 | 28 | -76 | 32 | |
| L Posterior Intraparietal Sulcus |  | 6.46 | -24 | -84 | 19 | |
| L Lateral Occipital Cortex, superior division (IPS) |  | 5.82 | -24 | -79 | 29 | |
| L Lateral Occipital Cortex, inferior division |  | 5.81 | -45 | -77 | -11 | |
| R Lateral Occipital Cortex, superior division (IPS) |  | 5.77 | 35 | -81 | 14 | |
| R Lateral Occipital Cortex, superior division (IPS) |  | 5.74 | 28 | -82 | 26 | |
| L Cuneal Cortex *(V3d)* |  | 5.65 | -5 | -81 | 18 | |
| L Lateral Occipital Cortex, inferior division |  | 5.56 | -46 | -77 | 14 | |
| R Inferior Temporal Gyrus, temporooccipital part |  | 5.53 | 49 | -60 | -16 | |
| R Lateral Occipital Cortex, superior division (IPS) |  | 5.52 | 23 | -83 | 36 | |
| R Lateral Occipital Cortex, inferior division |  | 5.39 | 47 | -71 | -12 | |
| R Lateral Occipital Cortex, inferior division |  | 5.38 | 38 | -83 | 5 | |
| L Lateral Occipital Cortex, inferior division |  | 5.32 | -51 | -72 | 10 | |
| L Lateral Occipital Cortex, superior division (IPS) |  | 5.27 | -22 | -85 | 36 | |
| R Lateral Occipital Cortex, inferior division |  | 5.25 | 45 | -74 | -9 | |
| R Lateral Occipital Cortex, inferior division |  | 5.17 | 48 | -65 | -15 | |
| R Precentral Gyrus | 2 246 | 4.67 | 39 | 9 | 28 | |
| R Middle Frontal Gyrus |  | 3.91 | 49 | 15 | 34 | |
| R Inferior Frontal Gyrus, pars opercularis |  | 3.51 | 53 | 13 | 24 | |
| R Precentral Gyrus *(lateral FEF)* | 1 251 | 4.56 | 40 | -2 | 57 | |
| R Precentral Gyrus *(lateral FEF)* |  | 3.84 | 40 | -2 | 47 | |
| R Middle Frontal Gyrus *(medial FEF)* |  | 3.31 | 30 | -3 | 63 | |
| *Note.* Voxel-level threshold was set to *p* < .001, uncorrected. Cluster-level threshold was set to *p* < .05, FWE-corrected. IPS = intraparietal sulcus. V3d = dorsal V3. FEF = frontal eye fields. The table lists all subpeaks extracted from SPM. | | | | | | |

| **Table S7**  *Results from regression analyses on first-level difference images (placebo-lorazepam) for combined pro- and antisaccades > baseline (extended version of Table 4)* | | | | | |  |
| --- | --- | --- | --- | --- | --- | --- |
| Anatomical label *(functional label)* | Cluster size | *t*-value | MNI coordinates | | | |
|  |  |  | x | y | z | |
| Predictor: Behavioral change scores for peak velocity (placebo-lorazepam); positive association | | | | | | |
| R Occipital Pole *(V1)* | 1 409 | 5.98 | 18 | -90 | 8 | |
| R Occipital Pole *(V3d)* |  | 5.33 | 23 | -93 | 12 | |
| R Occipital Pole |  | 5.10 | 34 | -90 | 12 | |
| R Precentral Gyrus *(medial FEF)* | 1 737 | 5.98 | 27 | -7 | 52 | |
| R Superior Frontal Gyrus *(medial FEF)* |  | 4.67 | 29 | -2 | 60 | |
| L Lateral Occipital Cortex, superior division (IPS) | 1 087 | 5.23 | -11 | -79 | 47 | |
| L Lateral Occipital Cortex, superior division (IPS) |  | 5.14 | -20 | -76 | 47 | |
| L Lateral Occipital Cortex, superior division (IPS) |  | 4.26 | -14 | -71 | 49 | |
| L Lateral Occipital Cortex, superior division (SPL) |  | 3.58 | -18 | -66 | 49 | |
| L Lateral Occipital Cortex, superior division (SPL) |  | 3.57 | -20 | -68 | 54 | |
| NA (L Cerebral White Matter) | 1 193 | 4.88 | -39 | 1 | 20 | |
| NA (L Cerebral White Matter) |  | 4.58 | -33 | -8 | 21 | |
| NA (L Cerebral White Matter) |  | 4.53 | -27 | -8 | 20 | |
| NA (L Cerebral White Matter) |  | 4.46 | -30 | -15 | 18 | |
| NA (L Cerebral White Matter) |  | 3.58 | -22 | -8 | 19 | |
| R Lateral Occipital Cortex, superior division (SPL) | 1 545 | 4.74 | 23 | -61 | 59 | |
| R Lateral Occipital Cortex, superior division (Area 7A, SPL) |  | 4.59 | 14 | -60 | 53 | |
| R Lateral Occipital Cortex, superior division (Area 7A, SPL) |  | 4.52 | 21 | -61 | 63 | |
| R Lateral Occipital Cortex, superior division (Area 7A, SPL) |  | 4.18 | 10 | -66 | 57 | |
| R Lateral Occipital Cortex, superior division (Area 7A, SPL) |  | 4.18 | 23 | -69 | 55 | |
| R Lateral Occipital Cortex, superior division (IPS) |  | 4.09 | 20 | -59 | 51 | |
| R Superior Parietal Lobule (IPS) |  | 3.89 | 22 | -56 | 53 | |
| R Lateral Occipital Cortex, superior division (SPL) |  | 3.80 | 18 | -75 | 53 | |
| Predictor: Change scores for lorazepam plasma level (placebo – lorazepam); negative association | | | | | | |
| NA (L Cerebral White Matter) | 7 924 | 6.03 | -26 | -52 | 29 | |
| L Cingulate Gyrus, posterior division |  | 5.97 | -7 | -41 | 26 | |
| L Precuneus |  | 5.69 | -14 | -56 | 27 | |
| NA (L Cerebral White Matter) |  | 5.50 | -29 | -50 | 27 | |
| NA (L Cerebral White Matter) |  | 5.48 | -17 | -53 | 31 | |
| L Precuneus |  | 5.43 | -15 | -52 | 23 | |
| L Planum Temporale (IPL) |  | 4.76 | -49 | -44 | 19 | |
| NA (L Cerebral White Matter) |  | 4.75 | -36 | -41 | 20 | |
| L Precuneus |  | 4.58 | -20 | -58 | 28 | |
| NA (L Cerebral White Matter) |  | 4.37 | -33 | -49 | 19 | |
| L Supramarginal Gyrus, posterior division (IPL) |  | 4.30 | -62 | -43 | 19 | |
| NA (L Cerebral White Matter) |  | 4.28 | -22 | -45 | 32 | |
| NA (L Cerebral White Matter) |  | 4.23 | -18 | -44 | 26 | |
| L Lateral Occipital Cortex, superior division (IPS) |  | 4.14 | -33 | -61 | 33 | |
| L Parietal Operculum Cortex (IPL) |  | 4.09 | -56 | -38 | 20 | |
| NA (L Cerebral White Matter) |  | 3.82 | -38 | -37 | 27 | |
| NA (R Cerebral White Matter) | 1 172 | 5.30 | 19 | -45 | 25 | |
| R Cingulate Gyrus, posterior division |  | 5.20 | 7 | -33 | 28 | |
| NA (R Cerebral White Matter) |  | 4.90 | 25 | -51 | 25 | |
| NA (R Cerebral White Matter) |  | 3.87 | 8 | -43 | 21 | |
| R Parietal Operculum Cortex | 2 423 | 5.14 | 40 | -21 | 17 | |
| R Central Opercular Cortex |  | 4.89 | 36 | -6 | 16 | |
| R Central Opercular Cortex |  | 4.67 | 42 | -12 | 14 | |
| R Insular Cortex |  | 4.53 | 35 | -12 | 16 | |
| R Central Opercular Cortex |  | 4.53 | 48 | -12 | 14 | |
| R Parietal Operculum Cortex |  | 4.11 | 53 | -21 | 14 | |
| R Insular Cortex |  | 3.53 | 32 | -17 | 7 | |
| NA (R Cerebral White Matter) |  | 3.52 | 31 | -15 | 11 | |
| R Parietal Operculum Cortex (IPL) |  | 3.39 | 59 | -25 | 18 | |
| L Cerebellum I-IV | 1 076 | 4.65 | -9 | -48 | -5 | |
| L Lingual Gyrus *(V2)* |  | 4.46 | -11 | -52 | -4 | |
| L Cerebellum I-IV |  | 4.29 | -5 | -52 | -1 | |
| L Cingulate Gyrus, posterior division |  | 3.79 | -6 | -48 | 5 | |
| L Lingual Gyrus (Subiculum) |  | 3.76 | -15 | -43 | -8 | |
| R Lingual Gyrus (Subiculum) | 1 825 | 4.63 | 13 | -48 | 1 | |
| R Precuneus *(V2)* |  | 4.62 | 14 | -58 | 13 | |
| R Parahippocampal Gyrus, posterior division |  | 4.46 | 12 | -38 | -3 | |
| L Precuneus *(V3d)* |  | 4.24 | -1 | -71 | 21 | |
| R Intracalcarine Cortex |  | 4.05 | 2 | -62 | 11 | |
| R Precuneus *(V2)* |  | 3.79 | 24 | -57 | 10 | |
| R Lingual Gyrus *(V1)* |  | 3.78 | 25 | -51 | -5 | |
| R Lingual Gyrus |  | 3.62 | 20 | -47 | -9 | |
| R Lingual Gyrus *(V2)* |  | 3.54 | 23 | -49 | 0 | |
| L Postcentral Gyrus | 1 980 | 4.62 | -49 | -17 | 43 | |
| L Postcentral Gyrus |  | 4.45 | -40 | -24 | 53 | |
| L Postcentral Gyrus |  | 4.40 | -44 | -21 | 53 | |
| L Precentral Gyrus |  | 4.27 | -37 | -24 | 50 | |
| L Precentral Gyrus |  | 3.89 | -28 | -21 | 72 | |
| L Precentral Gyrus |  | 3.79 | -34 | -23 | 69 | |
| L Postcentral Gyrus | 1 508 | 4.48 | -53 | -17 | 23 | |
| L Postcentral Gyrus (IPL) |  | 4.18 | -61 | -18 | 27 | |
| L Central Opercular Cortex |  | 3.89 | -47 | -15 | 18 | |
| L Precentral Gyrus |  | 3.88 | -58 | -5 | 20 | |
| L Central Opercular Cortex |  | 3.63 | -47 | -22 | 20 | |
| L Central Opercular Cortex |  | 3.62 | -43 | -22 | 19 | |
| L Central Opercular Cortex |  | 3.55 | -54 | -6 | 13 | |
| Predictor: Change scores for VAS contentedness (placebo – lorazepam); positive association | | | | | | |
| R Central Opercular Cortex | 1 165 | 4.94 | 47 | -1 | 1 | |
| R Insular Cortex |  | 4.45 | 43 | 12 | -9 | |
| R Planum Polare |  | 4.35 | 45 | -1 | -6 | |
| R Insular Cortex |  | 4.20 | 39 | 14 | -8 | |
| R Insular Cortex |  | 4.02 | 43 | 5 | -5 | |
| R Insular Cortex |  | 3.85 | 34 | 17 | -13 | |
| *Note. N* = 27 for peak velocity. *N* = 35 for plasma level. *N* = 36 for VAS contentedness. Voxel-level threshold was set to *p* < .001, uncorrected. Cluster-level threshold was set to *p* < .05, FWE-corrected. NA = not available (Neuromorphometrics atlas label). V3d = dorsal V3. Medial FEF = medial frontal eye fields. IPS = intraparietal sulcus. SPL = superior parietal lobule. IPL = inferior parietal lobule. The table lists all subpeaks extracted from SPM. | | | | | | |

**Figure S1**

*Brain activation for the main effect of saccade condition*


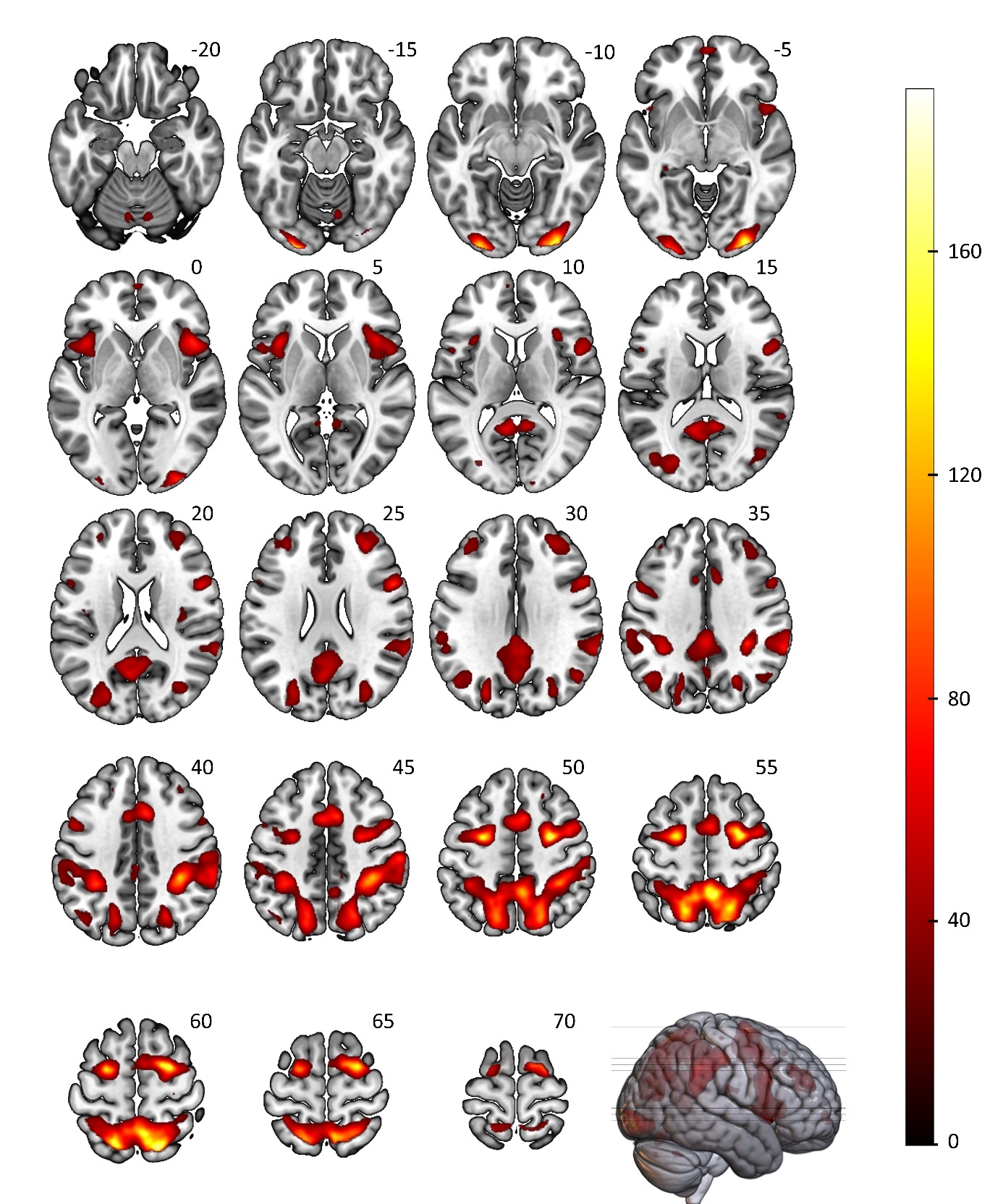


*Note.* Voxel-level threshold was set to *p* < .05, FWE-corrected. Cluster threshold was set to 20 voxels.

**Figure S2**

*Brain activation for the antisaccade > prosaccade contrast
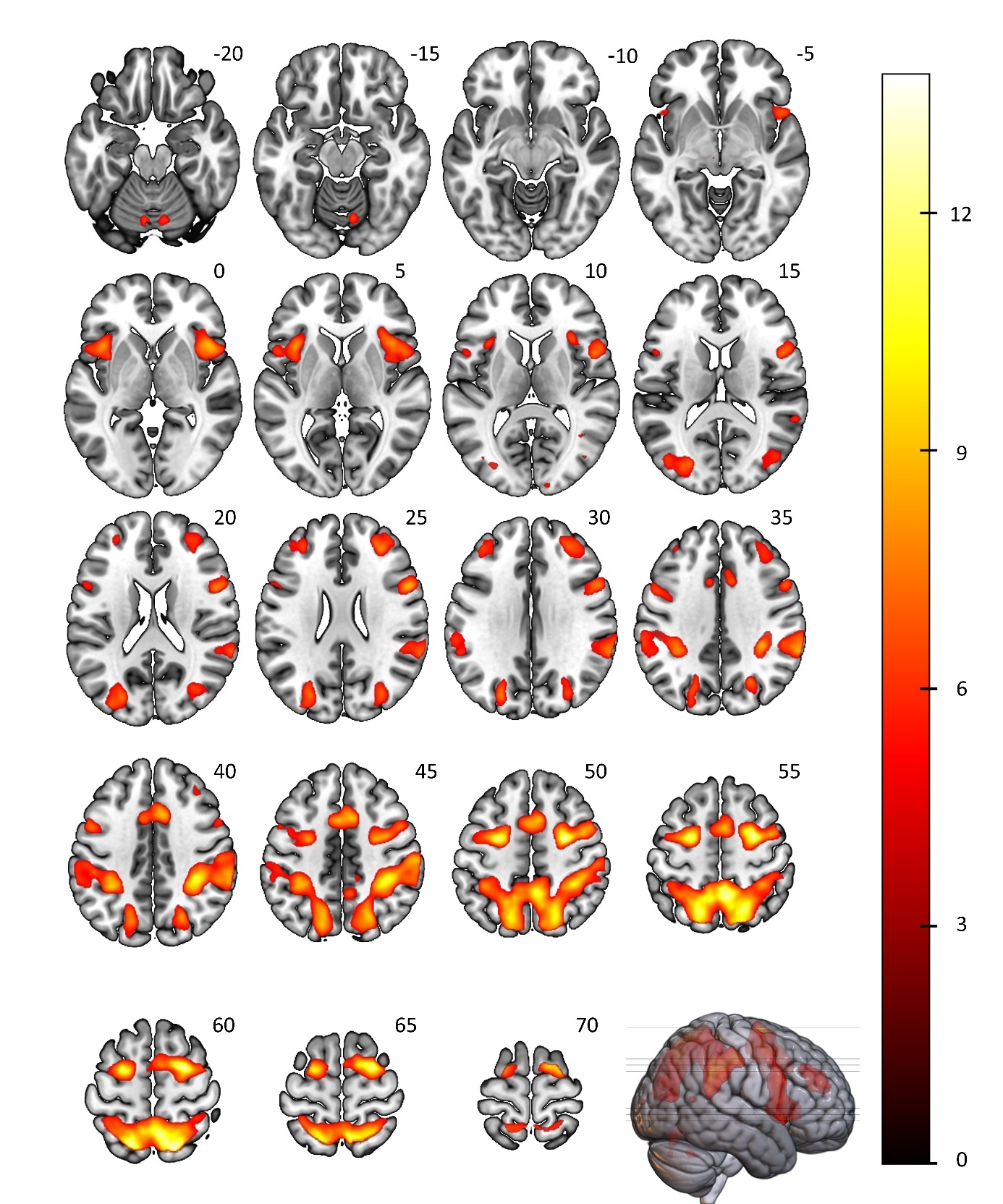
*

*Note.* Voxel-level threshold was set to *p* < .05, FWE-corrected. Cluster threshold was set to 20 voxels.

**Figure S3**

*Brain activation for the prosaccade > antisaccade contrast*

*
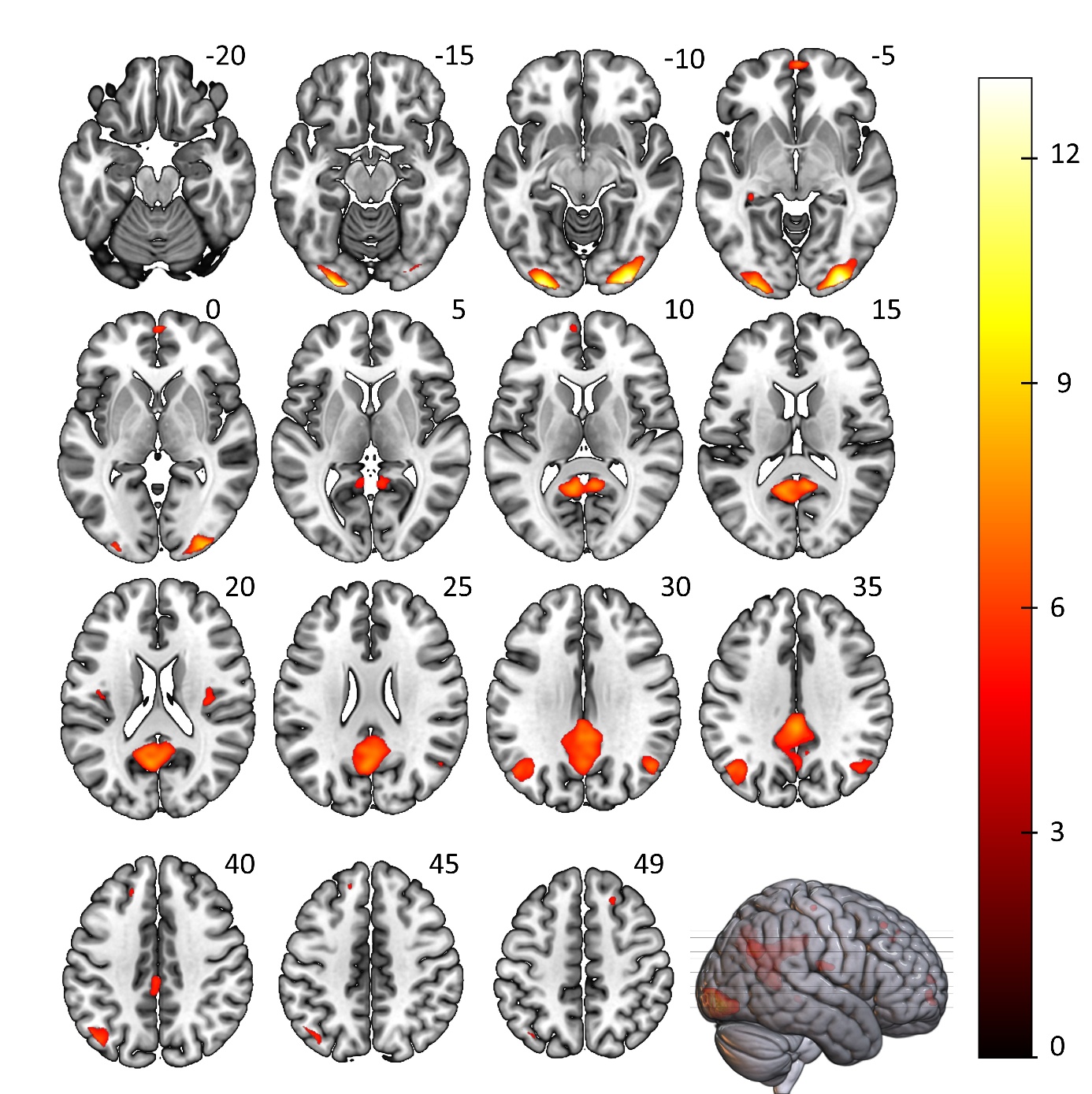
*

*Note.* Voxel-level threshold was set to *p* < .05, FWE-corrected. Cluster threshold was set to 20 voxels.

**Figure S4**

*GABA_A_ receptor density within the BZD-modulated network*

*
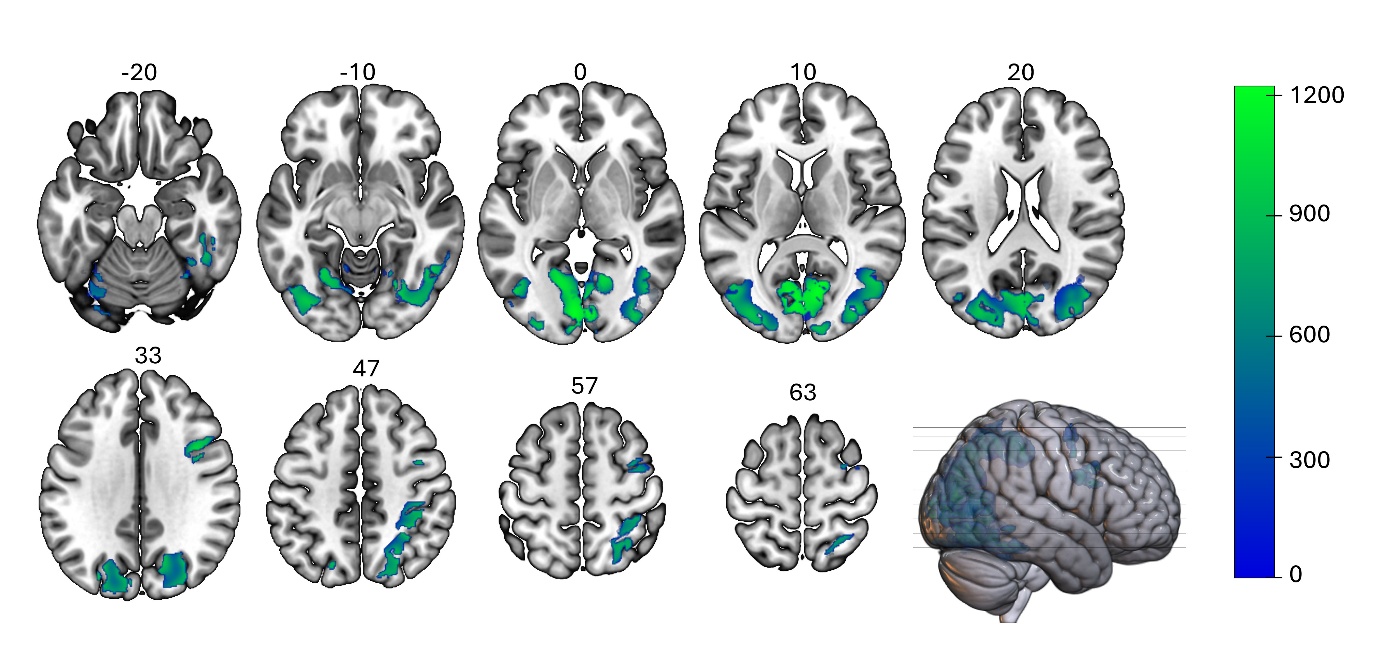
*

*Note.* Color bar indicates GABA_A_ receptor densities in pmol/ml. Depicted are the GABA_A_ receptor densities within the BZD-modulated network derived from the whole-brain analysis.

**References**

Ackenheil, M., Stotz-Ingenlath, G., Dietz-Bauer, R., Vossen, A., 1999. MINI international neuropsychiatric interview, German version 5.0.0, DSM IV. Psychiatrische Universitätsklinik München.

Aichert, D.S., Williams, S.C.R., Möller, H.J., Kumari, V., Ettinger, U., 2012. Functional neural correlates of psychometric schizotypy: An fMRI study of antisaccades. Psychophysiology 49, 345–356.

Ashburner, J., 2007. A fast diffeomorphic image registration algorithm. Neuroimage 38, 95–113.

Ashburner, J., Friston, K.J., 2005. Unified segmentation. Neuroimage 26, 839–851.

Bartra, O., McGuire, J.T., Kable, J.W., 2013. The valuation system: A coordinate-based meta-analysis of BOLD fMRI experiments examining neural correlates of subjective value. Neuroimage 76, 412–427.

Bates, D., Mächler, M., Bolker, B.M., Walker, S.C., 2015. Fitting linear mixed-effects models using lme4. J. Stat. Softw. 67, 1–48.

Behzadi, Y., Restom, K., Liau, J., Liu, T.T., 2007. A component based noise correction method (CompCor) for BOLD and perfusion based fMRI. Neuroimage 37, 90–101.

Bond, A., Lader, M., 1974. The use of analogue scales in rating subjective feelings. **Br. J. Med. Psychol.** 47, 211–218.

Chai, X.J., Castañón, A.N., Öngür, D., Whitfield-Gabrieli, S., 2012. Anticorrelations in resting state networks without global signal regression. Neuroimage 59, 1420–1428.

Ettinger, U., Ffytche, D.H., Kumari, V., Kathmann, N., Reuter, B., Zelaya, F., Williams, S.C.R., 2008. Decomposing the neural correlates of antisaccade eye movements using event-related fmri. Cereb. Cortex 18, 1148–1159.

Friston, K.J., Buechel, C., Fink, G.R., Morris, J., Rolls, E., Dolan, R.J., 1997. Psychophysiological and modulatory interactions in neuroimaging. Neuroimage 6, 218–229.

Hallquist, M.N., Hwang, K., Luna, B., 2013. The nuisance of nuisance regression: Spectral misspecification in a common approach to resting-state fMRI preprocessing reintroduces noise and obscures functional connectivity. Neuroimage 82, 208–225.

Jamadar, S.D., Fielding, J., Egan, G.F., 2013. Quantitative meta-analysis of fMRI and PET studies reveals consistent activation in fronto-striatal-parietal regions and cerebellum during antisaccades and prosaccades. Front. Psychol. 4, 749.

Kuznetsova, A., Brockhoff, P.B., Christensen, R.H.B., 2017. lmerTest package: Tests in linear mixed effects models. J. Stat. Softw. 82, 1–26.

Lancaster, J.L., Summerlin, J.L., Rainey, L., Freitas, C.S., Fox, P.T., 1997. The Talairach Daemon, a database server for Talairach atlas lables. Neuroimage 5, S633.

Lancaster, J.L., Woldorff, M.G., Parsons, L.M., Liotti, M., Freitas, C.S., Rainey, L., Kochunov, P. V., Nickerson, D., Mikiten, S.A., Fox, P.T., 2000. Automated Talairach atlas labels for functional brain mapping. Hum. Brain Mapp. 10, 120–131.

Maldjian, J.A., Laurienti, P.J., Burdette, J.H., 2004. Precentral gyrus discrepancy in electronic versions of the Talairach atlas. Neuroimage 21, 450–455.

Maldjian, J.A., Laurienti, P.J., Kraft, R.A., Burdette, J.H., 2003. An automated method for neuroanatomic and cytoarchitectonic atlas-based interrogation of fMRI data sets. Neuroimage 19, 1233–1239.

Martinez-Conde, S., Macknik, S.L., Troncoso, X.G., Hubel, D.H., 2009. Microsaccades: a neurophysiological analysis. Trends Neurosci. 32, 463–475.

McLaren, D.G., Ries, M.L., Xu, G., Johnson, S.C., 2012. A generalized form of context-dependent psychophysiological interactions (gPPI): A comparison to standard approaches. Neuroimage 61, 1277–1286.

Milner, A.D., Goodale, M.A., 1992. Separate visual pathways for perception and action. Trends Neurosci. 15, 20–25.

Nieto-Castanon, A., 2025. Preparing fMRI data for dtatistical analysis, in: Filippi, M. (Ed.), fMRI techniques and protocols . Springer, New York, pp. 163–191.

Nieto-Castanon, A., 2020. Handbook of functional connectivity magnetic resonance imaging methods in CONN. Hilbert Press, Boston, MA.

Nørgaard, M., Beliveau, V., Ganz, M., Svarer, C., Pinborg, L.H., Keller, S.H., Jensen, P.S., Greve, D.N., Knudsen, G.M., 2021. A high-resolution in vivo atlas of the human brain’s benzodiazepine binding site of GABA_A_ receptors. Neuroimage. 232, 117878.

Saari, T.I., Uusi-Oukari, M., Ahonen, J., Olkkola, K.T., 2011. Enhancement of GABAergic activity: Neuropharmacological effects of benzodiazepines and therapeutic use in anesthesiology. Pharmacol. Rev. 63, 243–267.

Sarmiento, L.F., Ríos-Flórez, J.A., Paez-Ardila, H.A., Lima de Sousa, P.S., Olivera-La Rosa, A., Oliveira da Silva, A.M.H., Gouveia, A., 2023. Pharmacological modulation of temporal discounting: A systematic review. Healthcare. 11, 1046.

Sheehan, D. V., Lecrubier, Y., Sheehan, K.H., Amorim, P., Janavs, J., Weiller, E., Hergueta, T., Baker, R., Dunbar, G.C., 1998. The Mini-International Neuropsychiatric Interview (MINI): The development and validation of a structured diagnostic psychiatric interview for DSM-IV and ICD-10. J. Clin. Psychiatry 59, 22–33.

Vossel, S., Geng, J.J., Fink, G.R., 2014. Dorsal and ventral attention systems: Distinct neural circuits but collaborative roles. Neuroscientist. 20, 150–159.

Whitfield-Gabrieli, S., Nieto-Castanon, A., 2012. Conn: A functional connectivity toolbox for correlated and anticorrelated brain networks. Brain Connect. 2, 125–141.

Worsley, K.J., Marrett, S., Neelin, P., Vandal, A.C., Friston, K.J., Evans, A.C., 1996. A unified statistical approach for determining significant signals in images of cerebral activation. Hum. Brain Mapp. 4, 58–73.
